# Healthy Eating Index, Epigenetic Age Acceleration and Mortality Risk in US Adults

**DOI:** 10.1111/acel.70504

**Published:** 2026-05-05

**Authors:** May A. Beydoun, Marie T. Fanelli Kuczmarski, Nicole Noren Hooten, Hind A. Beydoun, Jack Tsai, Ana I. Maldonado, Sharmin Hossain, Allen Nieva, Michele K. Evans, Alan B. Zonderman

**Affiliations:** ^1^ Laboratory of Epidemiology and Population Sciences National Institute on Aging, NIA/NIH/IRP Baltimore Maryland USA; ^2^ U.S. Department of Veterans Affairs VA National Center on Homelessness Among Veterans Washington DC USA; ^3^ Department of Management, Policy, and Community Health, School of Public Health University of Texas Health Science Center at Houston Houston Texas USA; ^4^ U.S. Department of Veterans Affairs Palo Alto California USA; ^5^ Department of Human Services State of Maryland Baltimore Maryland USA; ^6^ Department of Psychology Princeton University Princeton New Jersey USA

**Keywords:** additive Bayesian networks, biological aging, diet quality, epigenetic clocks, mortality

## Abstract

This study examined associations between diet quality, epigenetic age acceleration (EAA), and mortality in two U.S. cohorts: NHANES (*n* = 2158) and HRS (*n* = 1752), while accounting for demographic and socioeconomic (SES) determinants. Diet was evaluated as a potentially modifiable exposure within broader social and biological pathways. Participants were linked to the National Death Index. Cox proportional hazards models, additive Bayesian networks (ABN), generalized structural equation models (GSEM), and four‐way decomposition were used to estimate direct, indirect, and interaction effects, with SES specified as an upstream determinant of diet and aging processes. Higher diet quality (HEI‐2015) was associated with lower EAA and reduced mortality. GrimAgeEAA was the strongest mortality predictor (NHANES: HR = 1.61; HRS: HR = 1.76, both *p* < 0.001 per SD). ABN and GSEM identified SES as a driver of both diet and biological aging pathways. In HRS, approximately 44% of the inverse association between diet and mortality (HR = 0.85, *p* < 0.05) was explained by GrimAgeEAA (pure indirect effect), whereas mediation was not evident in NHANES. In sensitivity analyses adjusting for lifestyle factors and energy intake, the total effect in HRS was attenuated to non‐significance, with physical activity emerging as a key confounder. Diet quality also modified the association between PhenoAge and mortality (~22% attributable to interaction). Poor diet quality is associated with accelerated epigenetic aging and increased mortality risk, with patterns consistent with a modest mediating role of epigenetic mechanisms. However, these associations appear partly confounded by lifestyle factors, particularly physical activity, highlighting the importance of integrated behavioral and biological pathways.

## Graphical abstract

Higher diet quality (HEI‐2015) was associated with lower epigenetic age acceleration and reduced all‐cause mortality in NHANES and HRS, with GrimAgeEAA emerging as the strongest mortality predictor and partially accounting for the diet–mortality association in HRS, within a broader framework linking socioeconomic status, behavioral factors, and biological aging.

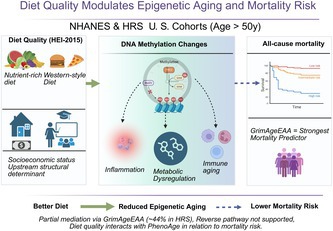

**Abbreviations**
ABN: additive bayesian networks
AGE: chronological age
AMPM: automated multiple‐pass method
BIC: bayesian information criterion
CBC: complete blood count
CDE/ereri_cde: controlled direct effect
CI: confidence interval
Cox model: Cox Proportional Hazards model
DAG: directed acyclic graph
DIED: death event
DNAm: DNA methylation
DunedinPACE: Dunedin pace of aging clock
DunedinPoAm: Dunedin pace of aging DNA methylation clock
EAA: epigenetic age acceleration
EFTF: enhanced face‐to‐face interview
ERERI: excess relative risk due to interaction
FDR: false discovery rate
FFQ: food frequency questionnaire
FNDDS: USDA food and nutrient database for dietary studies
FPED: food patterns equivalents database
GED: general educational development
GSEM: generalized structural equation modeling
HannumAgeEAA: hannum DNA methylation age acceleration
HCNS: health care and nutrition study
HEI‐2015: healthy eating index‐2015
HHS: U.S. department of health and human services
HISP: hispanic
HorvathAgeEAA: Horvath DNA methylation age acceleration
HR: Hazard ratio
HRS: health and retirement study
INTmed/ereri_intmed: mediated interaction
INTref/ereri_intref: reference interaction
IQR: interquartile range
IRB: institutional review board
Ln: natural logarithm
NCHS: National center for health statistics
NDI: National death index
NHANES: National health and nutrition examination survey
NHB: non‐hispanic black
NHW: non‐hispanic white
PhenoAgeEAA: phenotypic DNA methylation age acceleration
PIE/reri_pie: pure indirect effect
PoAm: pace of aging methylation
PSU: primary sampling unit
qPCR: quantitative polymerase chain reaction
RAND: research and development HRS dataset
RR: risk ratio
SD: standard deviation
SE: standard error
SES: socioeconomic status
TE/tereri: total effect/total excess relative risk
U.S.: United States
USDA: United States Department of Agriculture
WBC: white blood cell
Weibull model: Weibull Survival Regression model

## 1 Introduction

The quality of diet plays a fundamental role in shaping health outcomes, influencing both biological aging and longevity (Ohlhorst et al. 2013). Nutrient‐rich diets, characterized by adequately high consumption of fruits, vegetables, whole grains, and lean proteins, a pattern that characterizes the Mediterranean diet, have been linked to reduced risk of chronic diseases and extended lifespan (Tosti et al. 2018). Conversely, suboptimal eating patterns salient in the Western diet, which is marked by excessive intake of processed foods, refined sugars, and unhealthy fats, have been associated with increased risk of metabolic disorders, cardiovascular disease, and premature mortality (Clemente‐Suarez et al. 2023). An emerging area of research suggests that diet quality may also be associated with biological aging at the molecular level, as reflected by epigenetic age acceleration (EAA) (Bischoff‐Ferrari et al. 2025).

EAA, derived from DNA methylation patterns, provides an advanced measure of biological aging by capturing deviations between an individual's chronological age and their epigenetic age (Noroozi et al. 2021; Oblak et al. 2021). Greater EAA has been linked to adverse health outcomes, including heightened risk for cardiometabolic diseases, neurodegenerative disorders, and all‐cause mortality (Uchehara et al. 2023). Lifestyle factors such as nutrition, physical activity, smoking, and environmental exposures have been identified as key modulators of EAA, making it a valuable biomarker for understanding the long‐term effects of dietary habits on aging and health trajectories (Chervova et al. 2024; Oblak et al. 2021). While prior research has explored associations between specific dietary components—such as macronutrient composition, caloric intake, and adherence to dietary guidelines—and epigenetic aging, these studies have largely relied on traditional statistical approaches (Chiu et al. 2024; Kim et al. 2022; Li et al. 2024; Quach et al. 2017).

The interplay between diet quality, epigenetic mechanisms, and mortality is inherently complex, involving multiple direct and indirect pathways that cannot be fully captured using conventional regression models. These methods often assume linear relationships and fail to account for intricate dependencies among variables, potentially oversimplifying the true nature of diet‐epigenome‐mortality interactions. To address these limitations, this study employs additive Bayesian networks (ABN), a probabilistic graphical modeling approach that allows for the identification of complex interdependencies while incorporating both direct and indirect associations (https://r‐bayesian‐networks.org/) (Lewis and Ward 2013; Scutari and Denis 2019). Unlike standard regression techniques, ABN offers a data‐driven framework to model the structure of relationships among multiple variables, providing deeper insights into statistical dependencies and potential pathways consistent with mediation.

Using data from U.S. populations, we examined the links between diet quality, epigenetic age acceleration (EAA), and mortality. Our objectives were to: (1) assess the association between HEI‐2015 scores and multiple epigenetic clocks; (2) evaluate if EAA statistically accounts for (i.e., mediates) and/or statistically interacts with (i.e., moderates) the diet–mortality relationship using four‐way decomposition to partition effects into direct, indirect, and interaction components; while testing reverse causation as well and (3) elucidate complex pathways through ABN modeling. By integrating mediational analysis with machine learning, this study examines how dietary patterns are associated with biological aging and survival. These findings offer implications for precision nutrition strategies designed to promote longevity through targeted dietary interventions.

## 2 Materials and Methods

### 2.1 Databases

#### 2.1.1 National Health and Nutrition Surveys

The National Health and Nutrition Examination Survey (NHANES) consists of a series of cross‐sectional, nationally representative surveys conducted by the National Center for Health Statistics (NCHS) from the early 1970s (2013). Centers for Disease Control and Prevention (n.d.). In 1999, NHANES shifted to an ongoing series of biannual surveys (URL: https://wwwn.cdc.gov/nchs/nhanes/search/datapage.aspx?Component=Dietary&CycleBeginYear=1999). Essential body measurements were acquired through direct physical examination at a mobile assessment facility (Refer to Appendices I–III in S4 for specifics). Our investigation encompassed data from 1999 to 2002, associated with the death register until 2019. The NHANES data collection from 1999 to 2002 adhered to stringent ethical standards, encompassing informed consent, confidentiality, risk mitigation, and received approval from the NCHS.

#### 2.1.2 Health and Retirement Study

The Health and Retirement Study (HRS) is a longitudinal panel study that examines various factors affecting older Americans. HRS gathers data from a representative cohort of individuals aged 50 and above in the United States (URL: https://hrs.isr.umich.edu/about). The primary dataset includes variables gathered biennially from all HRS participants, covering various health and retirement‐related areas. The study uses the Research and Development (RAND) longitudinal dataset and the Enhanced Face‐to‐Face Interview (EFTF) to collect data on physical, biological, and psychosocial metrics. Off‐cycle data is also used to address specific factors, including biological markers of aging.

### 2.2 Mortality Linkage

NHANES provides linkage to mortality data with participants through the National Death Index (NDI), providing a mortality file to be integrated with demographic and other pertinent factors for each data wave. The HRS employs NDI linkage, interviews, and public records to monitor older persons, utilizing a tracker file that can be integrated with Core data and the RAND file, among others. The tracker file includes data on month and year of death up to the latest follow‐up date. The linkage with mortality is ongoing, rendering the study's design prospective cohort in nature.

### 2.3 Diet Quality: Healthy Eating Index‐15

The 2013 HRS Health Care and Nutrition Study (HCNS) aimed to measure, among other aspects, the dietary intake of elderly individuals utilizing data gathered through a food frequency questionnaire that evaluated the consumption of essential nutrients and food categories (Health and Retirement Study; Health and Retirement Study; Willett et al. 1985). The 2013 HCNS employed a validated Harvard Food Frequency Questionnaire (FFQ), initially created by Willett and associates, to evaluate the consumption of more than 100 food and beverage items over the preceding year (HRS 2013a; HRS 2013b; Willett et al. 1985). The survey included questions about food purchases and the usage of processed foods and beverages, such as sugary drinks, alcohol, and coffee. Food group and nutrient intake data collected with food frequency questionnaires can be used to calculate total and component scores for diet quality indices, including the Mediterranean Diet score and several iterations of the Healthy Eating Index (HEI) (HRS 2013a, 2013b; Hu et al. 1999; Lara‐Breitinger et al. 2023). In the current study, diet quality was assessed using the HEI‐2015. This index assesses the extent of conformity with the 2015–2020 Dietary Guidelines for Americans, encompassing 13 dietary components (NCI 2024; U.S. DHHS and USDA 2015). Component scores varied from 0 to 5 for total fruits, whole fruits, total vegetables, greens and beans, total protein foods, and seafood and plant proteins, while scores ranged from 0 to 10 for the other components, which included whole grains, dairy, fatty acids, refined grains, sodium, added sugars, and saturated fats. The HEI‐2015 possesses a maximum score of 100 points. A straightforward HEI scoring technique was employed (NCI 2024) (Refer to Appendix II S2 and the GitHub repository: https://github.com/baydounm/HRS_NHANES_HEIEPIGENMORT).

A three‐level variant of HEI‐2015 was utilized solely in a portion of the study (Kaplan–Meier curves and Log‐rank test), employing tertiles, whereas in the major part of our analyses, the continuous HEI‐2015 total score was incorporated into models as a standardized z‐score. A similar approach was applied to the NHANES 1999–2000 and 2001–2002, even though dietary assessment was accomplished using a single 24‐h recall (CDC 2024) USDA food codes that were elicited were merged with FPED data, to estimate food serving equivalents which were then summed up for each individual participant. The NHANES readily makes available nutrient intakes based on the single 24‐h recall. The combination of nutrients and food groups were used to calculate the total score of HEI‐2015, using a comparable script as for HRS (Beydoun, Georgescu, et al. 2025).

### 2.4 Socio‐Economic Status (SES) Index

Educational attainment and household income were combined using principal components analysis in both HRS and NHANES data. A single principal component was obtained and standardized into a z‐score. In HRS, educational attainment was categorized as “No degree”, “GED”, “High School graduate”, “Some college”, and “College degree or higher”; while household income/wealth was categorized as “< 25,000”, “25,000–125,999”, “125,000–299,999”, “300,000–649,999”, and “≥ 650,000”. The RAND HRS dataset calculates total household wealth by combining financial and non‐financial assets minus total debt. It uses multiple sources, including checking, savings, bonds, and IRAs. The total wealth is calculated using the RAND file, which is typically measured at the household level. Household total wealth in the current study was obtained from the 2012 HRS wave. In the NHANES, educational attainment, measured for adults above the age of 20 y, was grouped as “Less than 9th grade”, “9th–11th grade”, “High school graduate or GED equivalent”, “Some college of Associate's degree”, and “College graduate or higher”. In terms of household income, NHANES resorted to measuring poverty income ratio, which was used in our present study as a continuous variable. Values < 1 indicated below poverty household incomes, while values > 1 reflected an income that is above poverty levels.

### 2.5 Epigenetic Clocks

Both HRS and NHANES used Illumina Infinium MethylationEPIC BeadChip arrays to quantify DNA methylation. Each study used these data to calculate the epigenetic clocks as described for HRS (https://hrsdata.isr.umich.edu/data‐products/epigenetic‐clocks) and NHANES (https://wwwn.cdc.gov/nchs/nhanes/dnam/) These clocks included: Horvath 1, Hannum, Levine (PhenoAge), GrimAge, and Dunedin Pace of Aging (Belsky et al. 2020; Hannum et al. 2013; Horvath et al. 2016; Levine et al. 2018; Lu et al. 2019). Four clocks were transformed into EAA measures by regressing epigenetic age against chronological age and utilizing the residuals. The computation of EAA measures utilizing residuals was uniform across the two studies. More details are provided in Appendix III in S4.

### 2.6 Covariates

Our research used only specific demographics as exogenous factors, specifically self‐reported baseline age, sex (0 = Male, 1 = Female), and race/ethnicity. Race and ethnicity were harmonized whenever feasible, particularly between NHANES and HRS, leading to the establishment of categories for Non‐Hispanic White (NHW), Non‐Hispanic Black (NHB), and Hispanic, alongside a category for “Other ethnicities,” culminating in three dummy variables. In sophisticated studies of NHANES and HRS, NHW individuals were designated as the reference category for examining racial differences in mortality risk, biological aging, or both. As a sensitivity analysis, the four‐way decomposition models in both NHANES and HRS were additionally adjusted for white blood cell (WBC) composition, as described below. Adjustment focused on the largest available leukocyte subtypes expressed as percentages of total WBC. Owing to differences in data availability across cohorts, NHANES models included percentages of lymphocytes, monocytes, and neutrophils. In contrast, HRS models incorporated percentages of B cells, T cells, natural killer (NK) cells, monocytes, and dendritic cells. Additional methodological details are provided in Appendix VII in S4.

### 2.7 Study Samples

Figure S1 illustrates the participant flowcharts for the NHANES 1999–2002 and HRS 2016 samples. Both NHANES and HRS have epigenetic clock data for adults aged 50 and older. Figure S1 illustrates the progression from the initial RAND longitudinal dataset, which encompassed HRS and other prior data since 1992, to individuals aged 50 and above in the 2013 wave, subsequently to those with HEI‐2015 data collected in 2013, and ultimately to those possessing epigenetic clocks in the 2016 wave. The duration of follow‐up varied among cohorts, with NHANES exhibiting a lengthier follow‐up period of up to 20 years, in contrast to HRS, which had a maximum follow‐up of 7 years. The final sample for HRS was 1792, while the NHANES sample consisted of 2158 participants, with complete data on epigenetic clocks and HEI‐2015 among key covariates and aged 50+ years at baseline.

### 2.8 Statistical Methods

All analyses were conducted with Stata release 18.0 (StataCorp 2023), and visualizations were partially generated using R version 4.4.1 (R Core Team 2024). Initially, descriptive analyses detailed the distributions of essential variables, including means, medians, standard deviations, interquartile ranges, and frequency distributions for categorical data. Histograms and summary statistics (including means, standard deviations, and percentile distributions) were generated for each continuous variable to visually and analytically inspect distributional shape and identify extreme values. For all key continuous variables (e.g., HEI‐2015, SES index, epigenetic age acceleration measures, and age), outliers were evaluated using standardized criteria. Specifically, outliers were identified using a distribution‐based approach where values falling outside the range of 4 times the interquartile range (IQR) below the 25th percentile or above the 75th percentile were flagged, as was done a several previous studies using epigenetic and metabolomic data (Beydoun, Noren Hooten, et al. 2025; Beydoun, Weiss, et al. 2025; Qiang et al. 2024). We also conducted sensitivity analyses using alternative thresholds, which produced similar results. Extreme observations were handled by generating new variables that set these identified outliers to missing to ensure the robustness of the primary analyses. These procedures were applied uniformly to both NHANES and HRS datasets prior to model estimation to ensure comparability and minimize the influence of extreme observations on regression, survival, and network analyses.

Two cohorts of data were utilized, and descriptive statistics encompassed baseline characteristics and key variables across these cohorts, while considering the complexity of the sampling design to derive population estimates (including sampling weights, primary sampling units (PSUs), and strata for HRS and NHANES).

In the subsequent phase, Kaplan–Meier survival curves were generated for both cohorts, incorporating sampling weights to estimate survival probabilities over time while considering censored observations. Furthermore, these survival experiences were compared across tertiles of biological aging metrics and dietary quality (epigenetic DNAm age acceleration, HEI‐2015), and the statistical significance of differences in survival times was evaluated using Cox‐regression based tests, as was done in a previous study (Beydoun, Noren Hooten, et al. 2025). This segment of the analysis was modified to account for the complexity of the sampling design by using sampling weights.

Third, interconnections among biological aging indicators, SES, and diet quality were assessed using Pearson's correlations and displayed as heatmaps within each cohort. Sampling weights were not applied, as this analysis was descriptive and conducted to align methodologically with the ABN models, which do not accommodate survey weights.

Fourth, multivariable‐adjusted Cox proportional hazards models were performed following the assessment of hazard proportionality via Schoenfeld residuals. The models were calibrated for age, sex, race/ethnicity, and socioeconomic status (SES), with the primary exposures being the six biological aging metrics (i.e., epigenetic DNA methylation age acceleration) or dietary quality assessed using the HEI‐2015. The outcome measured was time to all‐cause mortality, with the analysis also adjusted for sampling weights.

Fifth, ABNs were used to model the joint dependency structure among demographics, SES, diet quality, multiple EAA measures described earlier, along with DunedinPoAm, in relation to mortality within a directed acyclic graph framework. This approach allows correlated biological aging markers to be evaluated simultaneously without prior data reduction, enabling the identification of the strongest conditional relationships rather than assuming that EAA measures biologically regulate one another. Mortality was modeled using a discrete‐time hazards framework based on person‐period data. Age, sex, and race/ethnicity were specified as exogenous; SES was allowed to be associated with diet as an antecedent variable; diet and SES were permitted to have directed edges toward EAA; and mortality was defined as the terminal outcome. These theory‐driven constraints preserved temporal plausibility while allowing clocks to “compete” within the network. Model fit was evaluated across one‐ to three‐parent‐per‐child structures, and the final model was selected based on log marginal likelihood and interpretability. Sampling weights were not incorporated due to current methodological limitations of ABN.

Ultimately, a Weibull regression modeling framework was employed for the mortality outcome, and generalized structural equations modeling (GSEM) was executed to replicate the final selected ABNs and estimate standard errors for each association identified in the final DAGs (Appendix VI in S4). This model was designed to assess the relationships among biological aging metrics, dietary quality, and mortality, as well as the pathways connecting age, sex, racial/ethnic, and SES differences with all‐cause mortality via dietary quality and biological aging metrics. This segment of the study was modified to account for the intricacies of the sampling design (sampling weights, primary sampling units, and strata) and was juxtaposed with a model that presumed simple random sampling. Thus, while ABN helped identify plausible directed pathways, GSEM allowed us to formally test those pathways, estimate standard errors, and account for the complex sampling design in NHANES and HRS.

Finally, a four‐way decomposition analysis was carried out to decompose the association between HEI‐2015 and all‐cause mortality into four components, namely, the controlled direct effect, reference interaction, mediated interaction, and pure indirect effect, through each epigenetic age acceleration (EAA) metric considered separately (HorvathAgeEAA, HannumAgeEAA, GrimAgeEAA, PhenoAgeEAA and DunedinPoAm) (Discacciati et al. 2018; VanderWeele 2014). Mortality risk was modeled using Cox proportional hazards regression, with time‐to‐event defined from baseline to death or censoring. For each EAA metric, a mediation–interaction framework consistent with counterfactual definitions was implemented, specifying HEI‐2015 as the exposure, the EAA measure as a candidate mediator within an exploratory framework, and mortality as the outcome. All models were adjusted for baseline demographic and socioeconomic covariates, including age, sex, race/ethnicity, education, and income‐to‐poverty ratio (or comparable SES indicators, depending on cohort). The mediator model (linear regression for continuous EAA) and the outcome model (Cox regression including exposure, mediator, and their interaction term) were jointly specified to estimate the four decomposition components on the hazard ratio scale. Proportion mediated and proportion attributable to interaction were derived from the decomposed effects. Sensitivity analyses included additional adjustment for white blood cell (WBC) composition as described earlier and Appendix VII S4, to account for potential confounding by immune cell heterogeneity in DNAm‐based aging measures. To assess potential bidirectionality, alternative models were specified in which EAA was treated as the exposure and HEI‐2015 as the mediator (EAA → HEI‐2015 → mortality), applying the same four‐way decomposition framework and covariate adjustment strategy. These analyses allowed evaluation of whether dietary quality mediated associations between accelerated biological aging and mortality risk, thereby probing the robustness and directionality of the hypothesized mediational pathway. These mediation analyses are exploratory and hypothesis‐generating and do not establish causal pathways due to the observational study design.

Two other sensitivity analyses were applied. First, in Cox models follow‐up of < 1 year was excluded and findings were compared with the full follow‐up period analysis. The second sensitivity analysis incorporated an inverse Mills ratio into the primary GSEM models, which were adjusted for sampling design complexity (See Appendix VIII in S4 and Tables SI in S4 and Table S2 in S5). The inverse Mills ratio was derived using a two‐stage Heckman selection procedure, in which a probit model estimated the probability of sample selection (1 = selected, 0 = not selected) as a function of age, sex, and race/ethnicity within each cohort, consistent with prior studies (Beydoun, Fanelli‐Kuczmarski, et al. 2018; Beydoun et al. 2020). Statistical significance was defined as a two‐sided type I error rate of less than 0.05. GSEM models were adjusted for multiple testing using the Benjamini–Hochberg false discovery rate (FDR) procedure, applied separately within each cohort and model, with statistical significance defined at *q* < 0.05.

## 3 Results

Table 1 presents the demographic, epigenetic, and mortality characteristics of the NHANES (1999–2002) and HRS (2013 and 2016) cohorts. Participants in the HRS cohort were older than those in NHANES (Mean (SE): 69.01 (0.3) vs. 63.9 (0.3) years, *p < 0.001*). The distribution of sex and race/ethnicity was similar across cohorts, with approximately 55% female participants and a majority identifying as NHW (~79%). The mortality rate per 1000 person‐years was lower in HRS (25.2, 95% CI: 22.1–28.8) than in NHANES (31.9, 95% CI: 29.4–34.6).

**TABLE 1:** Study characteristics and mortality risk across two cohorts (NHANES and HRS).

	NHANES 1999–2002	HRS 2013 and 2016
	Mean (SE) or %	Mean (SE) or %
Demographics	(*n* = 2158)	(*n* = 1752)
Age	63.9 (0.3)	69.01 (0.3)
Sex, % female	54.8	55.5
Race/ethnicity		
Non‐Hispanic White	79.3	79.8
Non‐Hispanic Black	8.2	8.6
Hispanic	9.1	8.3
Other	3.2	3.3
Epigenetic age acceleration metrics	(*n* = 2158)	(*N* = 1752)
HorvathAgeEAA	0.26 (0.19)	0.29 (0.21)
HannumAgeEAA	−0.19 (0.17)	0.12 (0.17)
PhenoAgeEAA	−0.21 (0.21)	−0.09 (0.23)
GrimAgeEAA	−0.35 (0.19)	−0.37 (0.14)
DunedinPoAm	1.10 (0.004)	1.07 (0.003)
SES z‐score	0.400 (0.047)	+0.179 (0.040)
HEI‐2015 z‐score	−0.015 (0.045)	−0.020 (0.024)
	(*N* = 2158)	(*N* = 1752)
Mortality rate, per 1000 person‐years, with 95% CI	31.9 (29.4–34.6)	25.2 (22.1–28.8)

Abbreviations: CI, confidence interval; DunedinPoAm, Dunedin pace of aging DNA methylation clock; GrimAgeEAA, grim DNA methylation epigenetic age acceleration; HannumAgeEAA, Hannum DNA methylation age; HEI‐2015, Healthy Eating Index, 2015 version; HorvathAgeEAA, Horvath DNA methylation age epigenetic age acceleration; HRS, Health and Retirement Study; *n*, unweighted sample size; NHANES, National Health and Nutrition Examination Surveys; PhenoAgeEAA, Pheno DNA methylation age epigenetic age acceleration; SE, standard error; SES, Socioeconomic status, based on educational attainment and income level.

**FIGURE 1:**
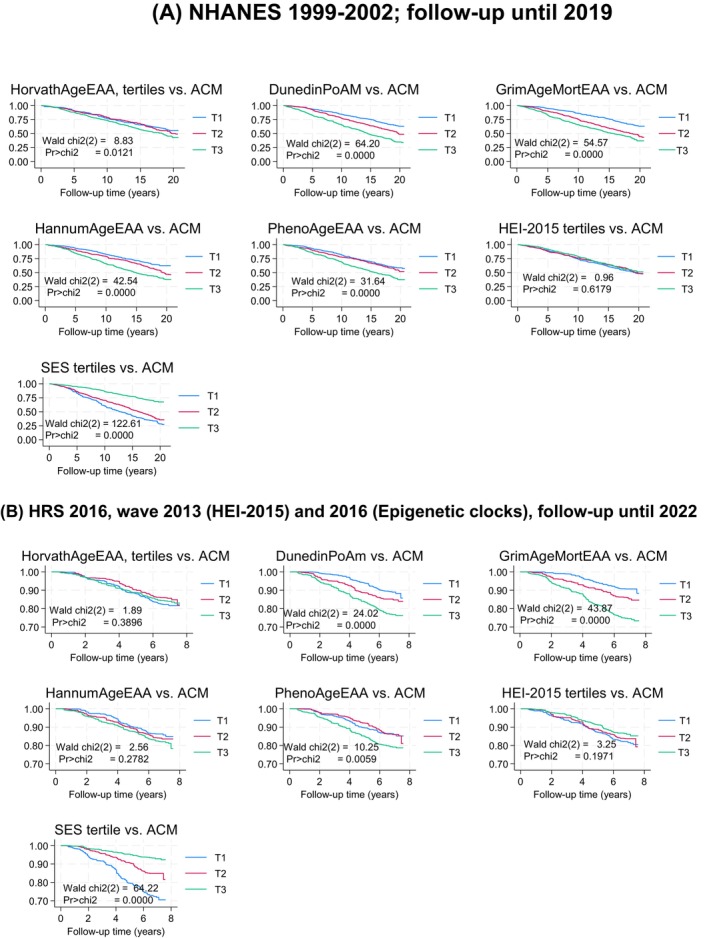
Kaplan–Meier survival curves across tertiles of SES, diet quality (HEI‐2015) and markers of biological aging for the s cohorts: NHANES 1999–2019 and HRS 2013 and 2016–2022. (A) NHANES 1999–2002; follow‐up until 2019. (B) HRS 2016, wave 2013 (HEI‐2015) and 2016 (Epigenetic clocks), follow‐up until 2022. Chi2 = Chi‐square; DunedinPoAm = Dunedin Pace of Aging DNA methylation clock; GrimAgeEAA = Grim DNA methylation Epigenetic Age Acceleration; HannumAgeEAA = Hannum DNA methylation Age, Epigenetic Age Acceleration; HEI‐2015 = Healthy Eating Index, 2015; HorvathAgeEAA = Horvath DNA methyalation Age, Epigenetic Age Acceleration; HRS=Health and Retirement Study; NHANES=National Health and Nutrition Examination Surveys; PhenoAgeEAA = Pheno DNA methylation Age Epigenetic Age Acceleration; SES=Socio‐economic Status; T1 = First tertile; T2 = Second Tertile; T3 = Third tertile. Kaplan–meier survival curves were conducted in both cohorts with time on study considered as the time variable to event (all‐cause death) or censoring by end of follow‐up. Maximum follow‐up time ranged from ~8 years for HRS to 20 years for NHANES. Median values for tertiles (T1/T2/T3) were 0.99 to 1.02/1.07 to 1.10/1.16 to 1.20 for DunedinPoAm across cohorts; −4.52 to −4.36/to −0.93 to −0.84/+4.26 to +4.68 for GrimAgeEAA; −4.58 to −4.47/+0.004 to +0.079/+4.45 to +4.48 for HannumAgeEAA; −4.20 to −4.74/ −0.109 to −0.570/4.42 to 5.59 for HorvathAgeEAA; −6.06 to −5.98/−0.53 to +0.04/6.01 to 6.07 for PhenoAgeEAA; HEI‐2010 and SES z‐scores have T1/T2/T3, corresponding approximately to −1/0/+1 for both cohorts. Sampling weights were accounted for in this analysis. Unweighted sample sizes were *n* = 2158 for NHANES and *n* = 1792 for HRS.

Kaplan–Meier survival analyses and Cox regression‐based tests were conducted to examine associations of SES, dietary and epigenetic factors with all‐cause mortality in two cohorts (Figure 1). In the NHANES 1999–2002 cohort (follow‐up until 2019), significant differences in survival were observed across groups for most comparisons (Wald *χ* ^2^ range: 8.83–122.61; *p*‐values < 0.05), except for one non‐significant result (*χ* ^2^ = 0.96; *p* = 0.6179) for HEI‐2015 tertiles. The HRS 2016 cohort, incorporating HEI‐2015 dietary scores (2013) and epigenetic aging measures (2016) with follow‐up until 2022, showed mixed findings. While some models demonstrated significant differences in survival (Wald *χ* ^2^ range: 10.25–64.22; *p*‐values < 0.05), others did not (*χ* ^2^ range: 1.89–3.25; *p*‐values > 0.05), mainly in the case of HorvathAgeEAA, HannumAgeEAA, and HEI‐2015 tertile. Overall, NHANES analyses yielded stronger and more consistent evidence of associations of SES and epigenetic factors with all‐cause mortality, whereas HRS findings indicated varying levels of significance depending on the model tested.

**FIGURE 2:**
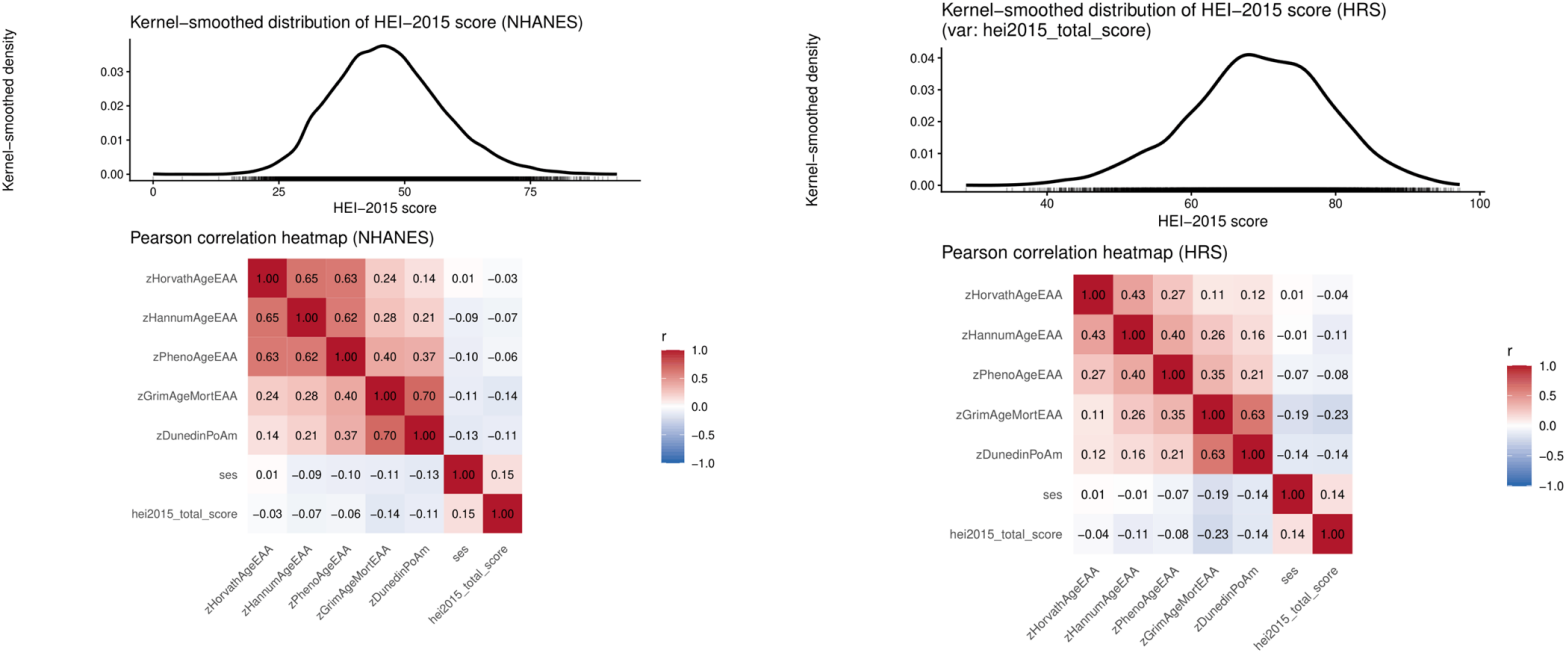
Pearson's correlation matrix between SES, diet quality (HEI‐2015) and epigenetic clock metrics: NHANES 1999–2002, HRS 2013 and 2016. DunedinPoAm = Dunedin Pace of Aging DNA methylation clock; GrimAgeEAA = Grim DNA methylation Epigenetic Age Acceleration; HannumAgeEAA = Hannum DNA methylation Age, Epigenetic Age Acceleration; HEI‐2015 = Healthy Eating Index, 2015; HorvathAgeEAA = Horvath DNA methyalation Age, Epigenetic Age Acceleration; HRS=Health and Retirement Study; NHANES=National Health and Nutrition Examination Surveys; PhenoAgeEAA = Pheno DNA methylation Age Epigenetic Age Acceleration; SES‐Socio‐economic Status; z = standardized z‐score. Sampling weights were not accounted for in this analysis. Unweighted sample sizes were *n* = 2158 for NHANES and *n* = 1792 for HRS.

Correlation heatmaps were generated to examine the associations among EAA factors, SES, and the HEI‐2015 in the NHANES 1999–2002 and HRS 2013 & 2016 cohorts (Figure 2). In both datasets, EAA measures exhibited moderate to strong correlations with the SES component score, suggesting a potential link between socio‐economic disadvantage and accelerated biological aging. Additionally, higher HEI‐2015 scores, indicative of better diet quality, were generally associated with lower epigenetic age acceleration, though the strength of associations varied across datasets. The NHANES heatmap revealed more consistent negative correlations between HEI‐2015 and EAA, whereas the HRS dataset showed weaker or more variable associations, potentially reflecting differences in population characteristics or measurement timing. Overall, the findings suggest that diet quality and SES may play important roles in shaping biological aging trajectories, especially in biological aging metrics, i.e., GrimAgeEAA and DunedinPoAM.

**FIGURE 3:**
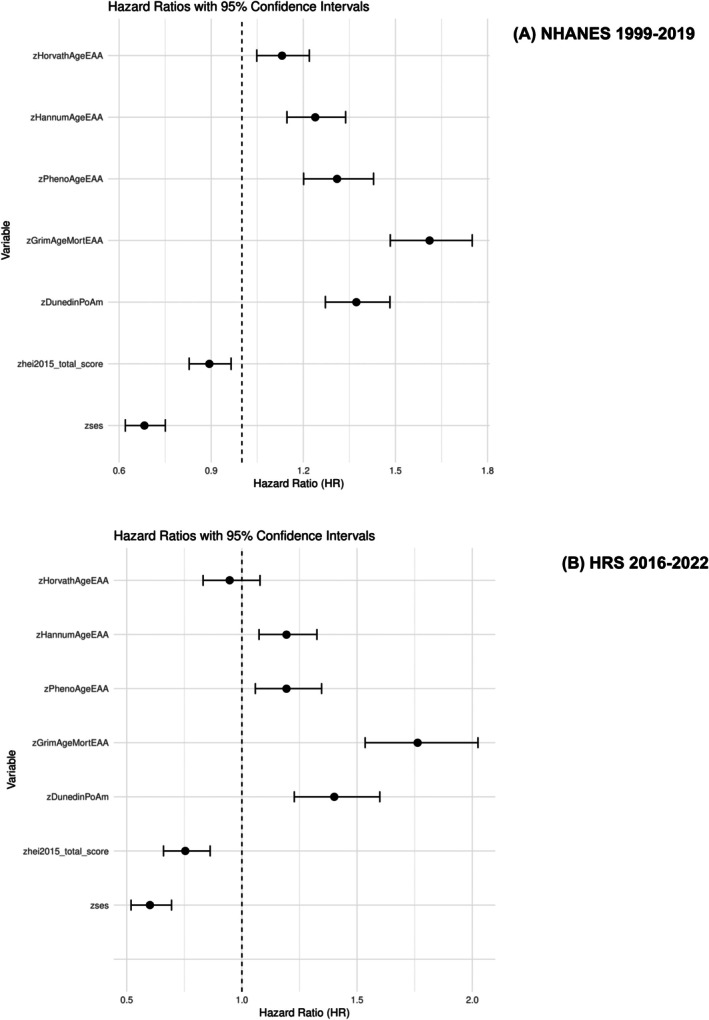
Association of each biological aging metric, diet quality and SES z‐scores with mortality risk adjusting for key exogenous variables: Cox proportional hazards models. DunedinPoAm = Dunedin Pace of Aging DNA methylation clock; GrimAgeEAA = Grim DNA methylation Epigenetic Age Acceleration; HannumAgeEAA = Hannum DNA methylation Age, Epigenetic Age Acceleration; HEI‐2015 = Healthy Eating Index, 2015; HorvathAgeEAA = Horvath DNA methyalation Age, Epigenetic Age Acceleration; HRS=Health and Retirement Study; NHANES=National Health and Nutrition Examination Surveys; PhenoAgeEAA = Pheno DNA methylation Age Epigenetic Age Acceleration; SES‐Socio‐economic Status; z = standardized z‐score. Models are adjusted for age, sex and race/ethnicity within each cohort. Values are Hazard ratios with 95% CI for each biological aging metric. Sampling weights were accounted for in this analysis. Unweighted sample sizes were *n* = 2158 for NHANES, *n* = 1792 for HRS.

Based on multi‐variable adjusted Cox proportional hazards models depicted in Figure 3, we examined the associations between key predictors and all‐cause mortality in both the NHANES 1999–2019 and HRS. The results showed significant associations between EAA, lower SES, and poorer diet quality, even after adjusting for demographic factors. Higher values of EAA were consistently associated with increased mortality risk (e.g., HR = 1.61, 95% CI:1.48–1.75, per standard deviation (SD) GrimAgeEAA in NHANES; HR = 1.76, 95% CI: 1.53–2.02, per SD of GrimAgeEAA in HRS). Lower SES z‐score was also linked to increased mortality (HR = 0.68, 95% CI:0.62–0.75, per SD of SES in NHANES; HR = 0.60, 95% CI:0.52–0.69 in HRS). Diet quality (HEI‐2015 scores) showed an inverse association with mortality risk, with higher scores reducing the hazard of death. The relationship between HEI‐2015 z‐scores and mortality was stronger in the HRS compared to NHANES (HR = 0.89, 95% CI:0.82–0.96, per SD of HEI‐2015 in NHANES; HR = 0.75, 95% CI:0.65–0.86, per SD of HEI‐2015 in HRS), while the overall findings suggest that these factors are associated with higher all‐cause mortality risk, even after adjusting for demographic factors. Exclusion of the first year of follow‐up from this analysis yielded comparable findings (See Github for sensitivity analysis results).

**FIGURE 4:**
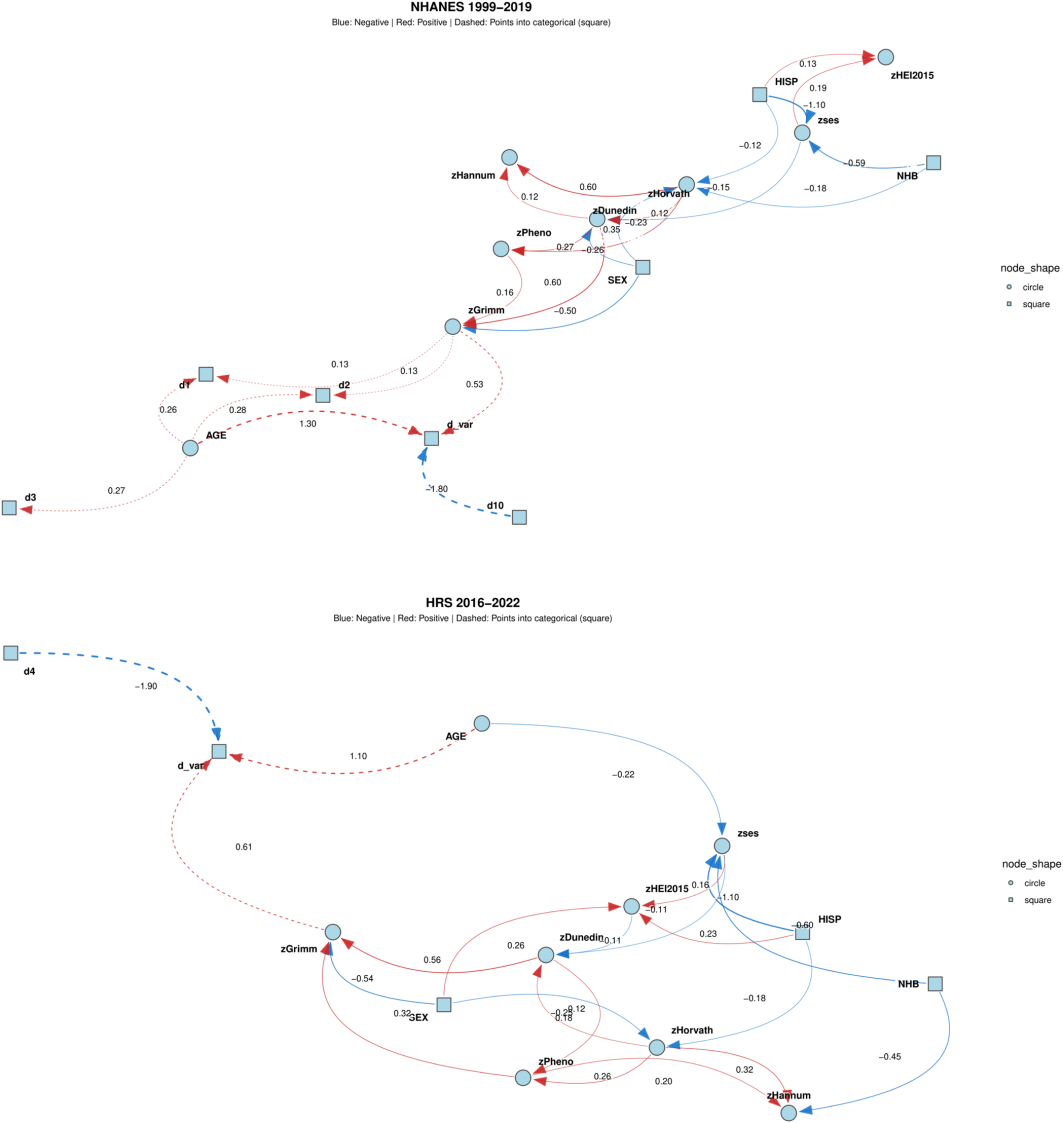
Additive Bayesian network solutions for 3 parents/child for associations among biological aging metrics, SES, HEI‐2015, demographics and mortality risk (discrete time hazards). AGE = chronological age; SEX = biological sex; NHB = non‐Hispanic Black; HISP = Hispanic; SES (zses) = standardized socioeconomic status score; HEI‐2015 (zHEI2015) = standardized Healthy Eating Index–2015 score; HorvathAgeEAA (zHorvath) = Horvath DNA methylation age acceleration; HannumAgeEAA (zHannum) = Hannum DNA methylation age acceleration; PhenoAgeEAA (zPheno) = phenotypic DNA methylation age acceleration; GrimAgeEAA (zGrimm) = GrimAge DNA methylation age acceleration; DunedinPoAm (zDunedin) = Dunedin Pace of Aging DNA methylation measure; d1, d2, d3, d4, d10 = discrete‐time mortality indicators across follow‐up intervals; d_var = overall mortality indicator; HRS = Health and Retirement Study; NHANES = National Health and Nutrition Examination Surveys; z = standardized z‐score. Additive Bayesian network (ABN) models depict conditional dependency structures among demographic factors, socioeconomic status, diet quality, epigenetic aging markers, and mortality using a directed acyclic graph framework. Nodes represent variables, with circular nodes indicating continuous variables and square nodes indicating categorical variables. Directed edges represent statistically derived conditional associations, with red lines indicating positive associations and blue lines indicating inverse associations. Dashed arrows denote relationships directed toward categorical outcomes. Edge labels represent local regression coefficients (linear or logistic, depending on node type). Mortality was modeled using a discrete‐time hazards framework, with d1–d10 (or d4 in HRS) representing time‐specific event indicators and d_var denoting the overall mortality outcome. Age, sex, and race/ethnicity were specified as exogenous variables; SES was modeled as an upstream determinant of diet and biological aging; and mortality was specified as the terminal node. The displayed networks correspond to the “3 parents per child” solution, selected based on model fit (log marginal likelihood) and interpretability relative to simpler structures. Models were estimated without sampling weights due to methodological constraints of ABN. Unweighted analytic sample sizes were *n* = 2158 for NHANES and *n* = 1792 for HRS.

Figure 4 compares findings from NHANES and HRS using the ABN “3 parents per child” solution, in which each non‐exogenous variable is allowed up to three antecedent predictors. In both cohorts, model fit improved as the number of parents per child increased, indicating a more complex and better‐fitting network structure relative to the 1‐ and 2‐parent solutions (Figure S2). Across cohorts, age and GrimAgeEAA emerged as the strongest direct predictors of binary mortality risk. Female sex was associated with lower mortality risk, primarily through pathways mediated by GrimAgeEAA alone or in combination with other epigenetic age acceleration (EAA) metrics. Non‐Hispanic Black (NHB) and Hispanic (HISP) participants, compared with NHW participants (reference), were more likely to have lower socioeconomic status (SES), and lower SES was in turn associated with poorer dietary quality—patterns observed consistently in both cohorts. SES was also inversely associated with biological aging as measured by DunedinPoAm, which showed a strong positive correlation with GrimAgeEAA, itself a direct predictor of mortality. In HRS only, higher diet quality was inversely related to DunedinPoAm, suggesting a pathway consistent with a potential link between diet to mortality through this biological aging marker and GrimAgeEAA. Overall, interrelationships among biological aging markers were moderately consistent across cohorts. Figure S2 presents the 1‐ and 2‐parent solutions alongside detailed results from the 3‐parent solution, illustrating that the simpler models yielded less informative networks regarding connections among epigenetic clocks and their links with age, sex, race/ethnicity, SES, diet quality, and the mortality outcome (d_var). The 3‐parent solution, summarized in Figure 4 and detailed in Figure S2, was subsequently carried forward for validation in the GSEM framework.

Findings of GSEM are presented in Table 2, using the final model obtained from ABN with the final equation being a Weibull model. Two models were used for each cohort, NHANES (1999–2019) and HRS (2016–2022): an unadjusted model for sampling design complexity and an adjusted model for sampling design complexity using weights, PSU, and strata. The results showed a strong positive association between baseline age and mortality in both cohorts. In NHANES, older chronological age was associated with higher HEI‐2015 scores and lower SES. Females had significantly lower values for EAA markers, such as HorvathAgeEAA, DunedinPoAm, and GrimAgeEAA. In HRS, females had a higher HEI‐2015 total score, which was in turn inversely related to the DunedinPoAm marker of biological aging. NHB individuals had significantly lower HannumAgeEAA. GrimAgeEAA was the strongest predictor of mortality in both cohorts; DunedinPoAm and PhenoAgeEAA were positively correlated with GrimAgeEAA, supporting their association with biological aging acceleration. Interrelations between epigenetic clocks were also positive in both cohorts. These findings were largely replicative of the ABN findings, particularly patterns consistent with potential mediation of DunedinPoAm and GrimAgeEAA in the association between HEI‐2015 and mortality risk. All results remained statistically significant upon adjustment for multiple testing using the Benjamini Hochberg (B‐H) procedure for FDR. Furthermore, inclusion of an inverse mills ratio in the GSEM models to adjust for potential selection bias did not alter our key findings (See Github for sensitivity analyses results).

**TABLE 2:** Generalized structural equations models in NHANES and HRS sample based on the 3‐parents/child limit Additive Bayesian Network Model solution for each cohort^a^.

	Model 1^b^		Model 2^c^	
	*β* (SE)	*p* ^d^	*β* (SE)	*p* ^d^
NHANES 1999–2019 (*n* = 2158)				
AGE➔DIED	+0.996 (0.034)	< 0.001	+1.087 (0.049)	< 0.001
AGE➔HEI‐2015	+0.093 (0.022)	< 0.001	+0.141 (0.032)	< 0.001
AGE➔SES	−0.243 (0.019)	< 0.001	−0.271 (0.021)	< 0.001
SEX➔HorvathAgeEAA	−0.196 (0.042)	< 0.001	−0.232 (0.056)	< 0.001
SEX➔DunedinPoAm	−0.267 (0.042)	< 0.001	−0.246 (0.053)	< 0.001
SEX➔GrimAgeEAA	−0.491 (0.027)	< 0.001	−0.420 (0.033)	< 0.001
NHB➔SES	−0.633 (0.050)	< 0.001	−0.633 (0.086)	< 0.001
NHB➔HorvathAgeEAA	−0.142 (0.055)	0.010	−0.151 (0.050)	0.005
HISP➔HEI‐2015	+0.149 (0.049)	0.002	+0.174 (0.061)	0.008
HISP➔SES	−1.052 (0.043)	< 0.001	−0.979 (0.073)	< 0.001
HISP➔HorvathAgeEAA	−0.116 (0.046)	0.014	−0.135 (0.085)	0.125
HISP➔HannumAgeEAA	+0.332 (0.032)	< 0.001	+0.217 (0.046)	< 0.001
HorvathAgeEAA➔DunedinPoAm	+0.132 (0.021)	< 0.001	+0.092 (0.037)	0.019
HorvathAgeEAA➔HannumAgeEAA	+0.628 (0.015)	< 0.001	+0.626 (0.043)	< 0.001
HorvathAgeEAA➔PhenoAgeEAA	+0.384 (0.019)	< 0.001	+0.389 (0.027)	< 0.001
HannumAgeEAA➔PhenoAgeEAA	+0.313 (0.020)	< 0.001	+0.297 (0.028)	< 0.001
PhenoAgeEAA➔GrimAgeEAA	+0.170 (0.015)	< 0.001	+0.169 (0.023)	< 0.001
GrimAgeEAA➔DIED	+0.428 (0.029)	< 0.001	+0.485 (0.042)	< 0.001
SES➔DunedinPoAm	−0.145 (0.020)	< 0.001	−0.200 (0.032)	< 0.001
SES➔HEI‐2015	+0.195 (0.024)	< 0.001	+0.261 (0.020)	< 0.001
DunedinPoAm➔GrimAgeEAA	+0.601 (0.015)	< 0.001	+0.640 (0.021)	< 0.001
DunedinPoAm➔PhenoAgeEAA	+0.246 ± 0.015	< 0.001	+0.240 (0.016)	< 0.001
HRS 2016–2022 (*n* = 1792)				
AGE➔DIED	+1.023 (0.061)	< 0.001	+1.070 (0.075)	< 0.001
AGE➔SES	−0.231 (0.022)	< 0.001	−0.253 (0.029)	< 0.001
SEX➔HEI‐2015	+0.281 (0.047)	< 0.001	+0.342 (0.054)	< 0.001
SEX➔HorvathAgeEAA	−0.263 (0.047)	< 0.001	−0.215 (0.057)	< 0.001
SEX➔GrimAgeEAA	−0.513 (0.034)	< 0.001	−0.533 (0.039)	< 0.001
NHB➔SES	−0.586 (0.062)	< 0.001	−0.608 (0.084)	< 0.001
NHB➔HannumAgeEAA	−0.443 (0.056)	< 0.001	−0.381 (0.069)	< 0.001
HISP➔HEI‐2015	+0.250 (0.073)	< 0.001	+0.176 (0.081)	0.034
HISP➔SES	−1.111 (0.067)	< 0.001	−1.167 (0.119)	< 0.001
HISP➔HorvathAgeEAA	−0.165 (0.071)	0.020	−0.238 (0.074)	0.002
HorvathAgeEAA➔HannumAgeEAA	+0.351 (0.021)	< 0.001	+0.351 (0.039)	< 0.001
HorvathAgeEAA➔DunedinPoAm	+0.112 (0.023)	< 0.001	+0.099 (0.033)	0.004
HorvathAgeEAA➔PhenoAgeEAA	+0.246 (0.023)	< 0.001	+0.236 (0.043)	< 0.001
PhenoAgeEAA➔HannumAgeEAA	+0.312 (0.021)	< 0.001	+0.303 (0.036)	< 0.001
PhenoAgeEAA➔GrimAgeEAA	+0.203 (0.017)	< 0.001	+0.210 (0.024)	< 0.001
GrimAgeEAA➔DIED	+0.561 (0.053)	< 0.001	+0.527 (0.060)	< 0.001
SES➔DunedinPoAm	−0.122 (0.024)	< 0.001	−0.144 (0.030)	< 0.001
SES➔HEI‐2015	+0.174 (0.024)	< 0.001	+0.179 (0.023)	< 0.001
HEI‐2015➔DunedinPoAm	−0.122 (0.024)	< 0.001	−0.154 (0.030)	< 0.001
DunedinPoAm➔GrimAgeEAA	+0.564 (0.018)	< 0.001	+0.582 (0.022)	< 0.001
DunedinPoAm➔PhenoAgeEAA	+0.186 (0.022)	< 0.001	+0.165 (0.030)	< 0.001

Abbreviations: AGE, baseline age; DIED, death event (yes vs. no); DunedinPoAm, Dunedin Pace of Aging DNA methylation clock; GrimAgeEAA, Grim DNA methylation epigenetic age acceleration; HannumAgeEAA, Hannum DNA methylation age, epigenetic age acceleration; HISP, hispanic; HorvathAgeEAA, Horvath DNA methylation age, epigenetic age acceleration; HRS, health and retirement study; *n*, unweighted sample size; NHANES, National Health and Nutrition Examination Surveys; NHB, non‐hispanic black; OTHER, other race/ethnicities; PhenoAgeEAA, Pheno DNA methylation age epigenetic age acceleration; SE, standard error; SEX, female vs. male.

^a^ Generalized structural equation models were conducted as a series of linear (most equations) and Weibull models (for the DIED outcome equation). The structure of each model was determined based on the 3‐parent limit solution from ABNs for NHANES and HRS cohorts.

^b^ Model 1 was conducted without adjustment for sampling design complexity and thus assumed a simple random sample.

^c^ Model 2 adjusted for sampling design complexity by including sampling weights, PSU, and strata that were most appropriate for each cohort.

^d^ *p*‐value for null hypothesis that path coefficient *β* = 0.

**FIGURE 5:**
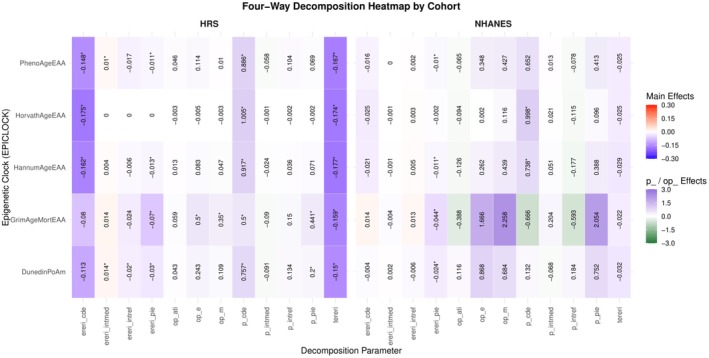
Heatmap of four‐way decomposition models for HEI‐2015 and all‐cause mortality by epigenetic age acceleration measures in NHANES (1999–2002, mortality follow‐up through 2019) and HRS (2013 dietary assessment; 2016 epigenetic assessment; mortality follow‐up through 2022). This figure displays standardized estimates from four‐way decomposition models evaluating epigenetic age acceleration (EAA) as a mediator of the association between diet quality (HEI‐2015, continuous z‐score) and all‐cause mortality. HEI‐2015 was modeled as the exposure, each EAA metric (HorvathAgeEAA, HannumAgeEAA, PhenoAgeEAA, GrimAgeEAA, and DunedinPoAm) was modeled separately as a continuous mediator using linear regression, and mortality was modeled using Cox proportional hazards regression. Rows represent epigenetic clocks and columns represent decomposition components. Color gradients reflect the magnitude and direction of effects (blue = inverse association; red = positive association). Cell values denote point estimates, with asterisks indicating statistical significance (*p* < 0.05). Decomposition parameters include: Tereri (total excess relative risk); ereri_cde (excess relative risk due to the controlled direct effect of HEI‐2015); ereri_pie (excess relative risk due to the pure indirect effect through EAA); ereri_intmed (excess relative risk due to mediated interaction); ereri_intref (excess relative risk due to reference interaction); terira (total effect risk ratio); proportion components (p_cde, p_pie, p_intmed, p_intref); and overall summary measures (op_m, overall proportion mediated; op_ati, overall proportion attributable to interaction; op_e, overall proportion eliminated if EAA were fixed). Models were adjusted for age, sex, race/ethnicity, and socioeconomic status. Unweighted analytic sample sizes were *n* = 2158 (NHANES) and *n* = 1752 (HRS).

Four‐way decomposition primary analyses, depicted in Figure 5, showed that higher HEI‐2015 was associated with lower mortality risk, but the total excess relative risk (tereri) was statistically significant only in HRS, not NHANES. In HRS, a meaningful portion of the inverse association was statistically attributed to EAA, particularly GrimAgeEAA, as reflected by significant pure indirect effects (44% of the total effect is PIE), while controlled direct effects remained present, indicating both mediated and independent pathways. In NHANES, indirect effects were directionally similar but weaker, consistent with the non‐significant total effect. Mediated and reference interaction components were generally small in both cohorts, suggesting limited interaction between HEI‐2015 and EAA (See Data S12, primary analysis).

Based on Figure S3 and Data [Link], [Link], [Link], reverse causation analyses (EAA → HEI‐2015 → mortality) provided little evidence that diet quality mediated the relationship between epigenetic aging and mortality. Although total effects of key clocks—particularly GrimAgeEAA—remained significant, the pure indirect effect through HEI‐2015 was small and generally non‐significant, with most of the excess risk attributable to the controlled direct effect. Interaction components were minimal overall. An exception was PhenoAge in HRS, where there was evidence of a pure interaction between HEI‐2015 and PhenoAge, accounting for approximately 25% of the total effect (largely driven by ~22% due to pure interaction). This moderating effect of diet persisted after adjustment for WBC composition. Adjustment for WBC composition in both the reverse and primary mediation models modestly attenuated estimates but did not materially change conclusions. In HRS, WBC adjustment reduced sample size and statistical power, slightly weakening previously observed mediating effects (from 1792 to 1153 participants with available data). Overall, findings are more consistent with a pathway in which diet is associated with mortality partly through epigenetic aging rather than the reverse.

Nevertheless, in a post hoc sensitivity analysis (Data S12, See Appendix IX in S4 for methods) applied to HRS data, adjusting for lifestyle factors (i.e., smoking status, alcohol use, physical activity) as well as energy intake attenuated the total effect of HEI on mortality to non‐significance, with physical activity emerging as the primary confounder. But, patterns remained consistent with primary HRS models, including modest but statistically significant mediation via GrimAgeEAA and significant HEI–PhenoAge interaction, supporting effect modification rather than mediation.

## 4 Discussion

### 4.1 Summary of Findings

This study examined how demographic factors, SES, diet quality, and EAA relate to all‐cause mortality in NHANES and HRS. Lower SES was associated with higher biological aging, and better diet quality was generally linked to lower EAA, particularly in HRS. Cox, ABN, and GSEM models consistently identified age and GrimAgeEAA as the strongest predictors of mortality. In HRS, the inverse association between diet quality and mortality was statistically significant, with approximately 44% accounted for by GrimAgeEAA. However, incorporating lifestyle factors and energy intake attenuated this total effect to non‐significance, with physical activity emerging as the primary confounder. Despite this attenuation, mediation through GrimAgeEAA remained modest but statistically significant, and PhenoAge continued to exhibit a significant interaction with diet quality.

### 4.2 Previous Studies

#### 4.2.1 Epigenetic Clocks, Morbidity and Mortality

DNA methylation based epigenetic clocks have been utilized to predict age‐associated disorders and mortality (Fransquet et al. 2019; Horvath and Raj 2018). A meta‐analysis of 23 studies revealed that a 5‐year rise in DNA methylation age correlates with an 8%–15% elevation in all‐cause mortality risk (Fransquet et al. 2019). Furthermore, intrinsic EAA Hannum and GrimAgeEAA forecasted oropharyngeal cancer mortality, overall cancer mortality, and cardiovascular mortality (Beynon et al. 2022; Mendy and Mersha 2024). In our study, GrimAgeEAA was the strongest predictor of all‐cause mortality in both cohorts. Other EAA measures were also associated with mortality, mainly in the NHANES study. These data are consistent with the fact that the GrimAge epigenetic clock was created by identifying DNA methylation sites that were associated with plasma proteins, then creating a composite of DNA methylation‐based surrogate biomarkers that were associated with time to death. These findings suggest that epigenetic measures, in particular GrimAge, can be used to predict mortality.

#### 4.2.2 Association of Socio‐Demographic and Economic Factors With Dietary Quality

Prior work underscores the strong relationship of SES factors with diet quality across different populations, life stages, and geographic regions. Consistent with our current findings, previous studies have found that higher SES—whether measured by income, education, or occupation—is linked to better diet quality (Arabshahi et al. 2011; Beghin et al. 2014; Beydoun and Wang 2008; Cabrera et al. 2007; Ryden and Hagfors 2011). This relationship is observed in various age groups, from adolescents (Beghin et al. 2014) to older adults (Cabrera et al. 2007), indicating that nutritional differences by SES persist throughout the lifespan. Nutrition knowledge and beliefs serve as potential moderators of this relationship, as individuals with greater awareness of healthy eating habits tend to make better dietary choices regardless of SES (Beydoun and Wang 2008). However, even when awareness is present, economic barriers may still limit access to high‐quality diets, as evidenced by studies showing that healthier diets tend to be more expensive (Beydoun et al. 2015; Beydoun, Hossain, et al. 2018; Ryden and Hagfors 2011). These financial constraints particularly impact lower‐income groups, exacerbating dietary differences. Another common theme found in recent studies is the role of lifestyle factors in shaping diet quality (Arabshahi et al. 2011; Lioret et al. 2014). These findings suggest that diet quality is not only shaped by SES but also by broader behavioral and environmental influences.

#### 4.2.3 Association of Dietary Quality With Epigenetic Clocks

Furthermore, research also highlights the significant influence of diet, lifestyle, and metabolic health on epigenetic aging (Biemans et al. 2024; Chiu et al. 2024; Dwaraka et al. 2024; Dye et al. 2024; Grootswagers et al. 2024; Kim et al. 2022; Li et al. 2024; Quach et al. 2017; Sharma and Bhonde 2023; Thomas et al. 2024; Thomas et al. 2023). High‐quality diets rich in plant‐based foods and essential nutrients slow epigenetic aging, while poor diets with added sugars accelerate it (Biemans et al. 2024; Grootswagers et al. 2024). Consistently, research has demonstrated that high diet quality, characterized by plant‐based foods, lean meats, and essential nutrients, is associated with a deceleration of epigenetic aging (Chiu et al. 2024; Dwaraka et al. 2024; Kim et al. 2022; Quach et al. 2017). In contrast, increased consumption of added sugars and poor dietary quality accelerate epigenetic aging, reinforcing the importance of nutrient‐dense diets in promoting longevity (Biemans et al. 2024; Chiu et al. 2024). Similarly, obesity and metabolic dysfunction contribute to faster biological aging, though the effects of weight loss remain unclear (Li et al. 2024; Quach et al. 2017). Lifestyle factors like physical activity and education offer protective benefits, emphasizing the multifactorial nature of aging (Quach et al. 2017). Emerging research suggests that dietary and molecular interventions may help mitigate age‐related diseases and cognitive decline, underscoring the need for further exploration of mechanistic pathways and long‐term effects (Sharma and Bhonde 2023; Thomas et al. 2024).

#### 4.2.4 Dietary Quality, Morbidity, and Mortality

Similarly, prior research has associated dietary indices with health outcomes, indicating that poor diets represent a modifiable risk factor for non‐communicable illnesses and mortality (Brlek and Gregoric 2023). Recent cohort studies indicate that reduced dietary acidity and inflammatory food components, together with adherence to a high‐quality diet, may mitigate cardiovascular disease and death (Fereidouni et al. 2023; Kang et al. 2023). An increased plant‐based diet index over time shown a 10% reduction in total mortality risk. A nutritious diet, along with beneficial lifestyle factors such as physical activity, can diminish mortality rates, cardiovascular disease, and cancer risk, while also offering protection for pre‐frail and frail elderly individuals (Brown et al. 2016; Ding et al. 2022). Subpar food quality in adult populations correlates with heightened mortality risk, and evaluating diet quality by dietary indices may indirectly predict other health determinants, including frailty, cardiovascular disease, and mortality (Brlek and Gregoric 2023; Gicevic et al. 2021; Jayanama et al. 2021).

### 4.3 Potential Mechanistic Explanations for Our Findings

One important finding is the interaction between diet quality and PhenoAge in relation to mortality risk among the older adults participating in HRS. A plausible mechanism is that PhenoAge reflects inflammatory and cardiometabolic dysregulation, and diet quality modifies how strongly this phenotype translates into mortality risk. PhenoAge incorporates DNAm surrogates of clinical markers such as CRP, glucose, albumin, creatinine, and white blood cell count, capturing systemic inflammation and metabolic strain (Levine et al. 2018). These biomarkers are well‐established predictors of morbidity and mortality and reflect pathways central to cardiometabolic and immune aging (Ferrucci and Fabbri 2018). Poor diet quality may exacerbate inflammatory signaling and metabolic dysregulation, whereas healthier dietary patterns reduce CRP, improve glycemic control, and enhance metabolic resilience, thereby attenuating mortality risk among individuals with elevated PhenoAge (Estruch et al. 2013; Schwingshackl et al. 2017).

In parallel, diet may be associated with mortality through biological aging pathways reflected in DunedinPoAm and GrimAgeEAA. DunedinPoAm captures the pace of aging through DNAm changes linked to multisystem decline, including immune and metabolic regulation (Belsky et al. 2020; Belsky et al. 2022). GrimAgeEAA incorporates DNAm surrogates of plasma proteins such as plasminogen activator inhibitor‐1 (PAI‐1), growth differentiation factor‐15 (GDF‐15), leptin, and smoking pack‐years—markers strongly tied to vascular dysfunction, inflammation, and mortality (Lu et al. 2019). Healthy dietary patterns, such as the Mediterranean and DASH diets, are often found to be inversely related to oxidative stress and systemic inflammation (Aleksandrova et al. 2021). These diets may provide essential nutrients that neutralize reactive oxygen species and dampen pro‐inflammatory signaling pathways, such as NF‐κB (Tan et al. 2018). Such nutritional interventions can modulate DNA methylation patterns, effectively slowing epigenetic aging markers like GrimAge and DunedinPoAm (Kim et al. 2022; Quach et al. 2017). By regulating the expression of genes involved in metabolic and inflammatory homeostasis, high‐quality diets reduce the biological aging rate (Maugeri and Barchitta 2020). Ultimately, these epigenetic patterns may statistically account for a portion of the observed association of diet on all‐cause and cardiovascular mortality (Wang et al. 2023), as is re‐enforced by our present study.

### 4.4 Strengths and Limitations

This study leverages two nationally representative U.S. cohorts with linked mortality data, allowing for independent cross‐setting comparisons. Utilizing standardized HEI‐2015 scores alongside multiple epigenetic clocks captures biological aging from complementary perspectives. Furthermore, integrating ABN and GSEM with survival models coupled with four‐way decomposition models clarifies the complex interconnections between socioeconomic, dietary, and biological factors.

Interpretations are limited by issues of temporality ascertainment and various other potential biases. In HRS, a 3‐year gap between diet (2013) and epigenetic assessment (2016) prevents definitive temporal ordering, and survival‐related selection may bias estimates. In NHANES, contemporaneous measurement and the use of single 24‐h recalls—subject to random measurement error—may bias associations toward the null. Across both cohorts, single‐point epigenetic measures prevent the assessment of longitudinal biological change. Differing dietary instruments (FFQ in HRS vs. 24‐h recall in NHANES), age distributions, and follow‐up durations complicate direct effect‐size comparisons. Additionally, ABN remains exploratory and does not establish causality, leaving potential for residual confounding from unmeasured health behaviors or comorbidities. Therefore, mediation results, specifically diet's indirect inverse association on mortality via GrimAgeEAA and DunedinPoAm, should be interpreted as exploratory and hypothesis‐generating rather than evidence of causal mediation. Four‐way decomposition relies on four rigorous “no‐confounding” assumptions to ensure there are no unmeasured factors influencing the exposure, mediator, or outcome relationships (Discacciati et al. 2018; VanderWeele 2014). Additionally, it requires consistency, composition, and positivity to ensure that potential outcomes are correctly identified and every treatment level is represented across the data (Discacciati et al. 2018; VanderWeele 2014). Future longitudinal research with repeated assessments is required to confirm causal pathways. Nonetheless, consistent patterns across cohorts and methods reinforce the link between socioeconomic disadvantage, poor diet, and accelerated biological aging.

## 5 Conclusions

In summary, poor diet quality is linked to accelerated biological aging and increased mortality, with GrimAgeEAA serving as the strongest predictor across cohorts. In HRS, a substantial portion of the diet–mortality association was statistically accounted for by epigenetic mechanisms, but attenuated after accounting for physical activity, suggesting residual confounding. Nevertheless, consistent mediation patterns through GrimAgeEAA and interaction effects involving PhenoAge support a framework in which diet operates within a broader network of behavioral and biological processes influencing survival. These findings should be interpreted as hypothesis‐generating and warrant confirmation in longitudinal studies with repeated measures.

## Author Contributions

**May A. Beydoun:** conceptualization, data curation, statistical analysis, supervision, data acquisition, methodology, validation, manuscript drafting, and revision. **Marie T. Fanelli Kuczmarski:** conceptualization, methodology, validation, manuscript drafting, and revision. **Nicole Noren Hooten:** conceptualization, data acquisition, methodology, resources, validation, manuscript drafting, and revision. **Hind A. Beydoun:** conceptualization, data curation, manuscript drafting, and revision. **Jack Tsai:** conceptualization, data acquisition, methodology, resources, validation, manuscript drafting, and revision. **Ana I. Maldonado:** conceptualization, data curation, manuscript drafting, and revision. **Sharmin Hossain:** conceptualization, data curation, manuscript drafting, and revision. **Allen Nieva:** conceptualization, data curation, manuscript drafting, and revision. **Michele K. Evans:** conceptualization, supervision, data acquisition, resources, manuscript drafting, and revision. **Alan B. Zonderman:** conceptualization, data curation, supervision, data acquisition, resources, manuscript drafting, and revision. All authors read and approved the final manuscript.

## Funding

This work was supported by the Intramural Research Program of the National Institute on Aging, National Institutes of Health (NIA/NIH/IRP) under project number AG000513.

## Disclosure

The authors have nothing to report.

## Conflicts of Interest

The authors declare no conflicts of interest.

## Supporting information


**FIGURE S1:** Participant flowcharts for NHANES, HRS and HANDLS samples.

**FIGURE S2:** ABN findings using discrete time hazards models, for 1–3 parents/child limits.

**FIGURE S3:** Combined heatmaps of four‐way decomposition models across primary and sensitivity analyses.

**Appendices:** Comparison of Included vs. Excluded Participants and Selection Model for Analytical Samples (NHANES 1999–2002 and HRS 2013–2016) Panel A. Included vs. Excluded.

**TABLE S2:** Full generalized structural equation models (GSEM) including inverse Mills ratio (IMR) in selection‐bias.

**DATA S1:** acel70504‐sup‐0006‐DataS1.xlsx.

**DATA S2:** acel70504‐sup‐0007‐DataS2.xlsx.

**DATA S3:** acel70504‐sup‐0008‐DataS3.xlsx.

**DATA S4:** acel70504‐sup‐0009‐DataS4.xlsx.

**DATA S5:** acel70504‐sup‐0010‐DataS5.xlsx.

**DATA S6:** acel70504‐sup‐0011‐DataS6.xlsx.

**DATA S7:** acel70504‐sup‐0012‐DataS7.xlsx.

## Data Availability

NHANES and HRS data are publicly available at: https://www.cdc.gov/nchs/nhanes/?CDC_AAref_Val=https://www.cdc.gov/nchs/nhanes/index.htm https://www.cdc.gov/nchs/nhanes/index.htm and https://hrs.isr.umich.edu/about, respectively. Code and analytic outputs supporting the findings of this study are available from the corresponding author upon reasonable request and will be made publicly available at: https://github.com/baydounm/HRS_NHANES_HEIEPIGENMORT.
