# Supplementary material for: Healthy Eating Index, Epigenetic Age Acceleration and Mortality Risk in US Adults
Source: Aging Cell. 2026 May 5;25(5):e70504. doi: 10.1111/acel.70504 (PMC13143866; doi:10.1111/acel.70504)
Supplement: Supplementary file 1 — FIGURE S1: Participant flowcharts for NHANES, HRS and HANDLS samples. [file ACEL-25-e70504-s003.pdf]

FIGURE S1. Participant flowcharts for NHANES, HRS and HANDLS samples

(A) NHANES 1999-2002

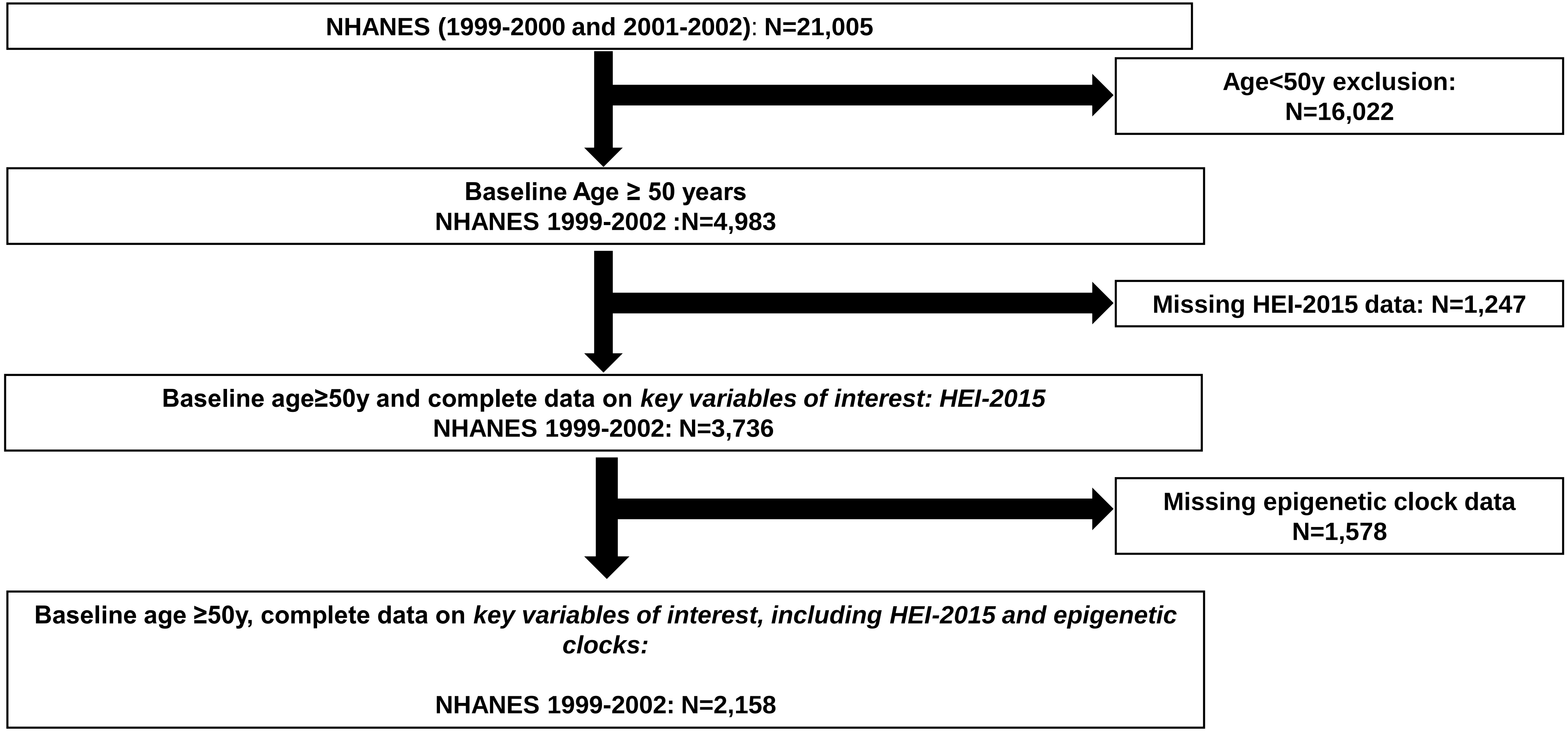

(B) HRS 2013 (HEI-2015) and 2016 (epigenetic clocks)

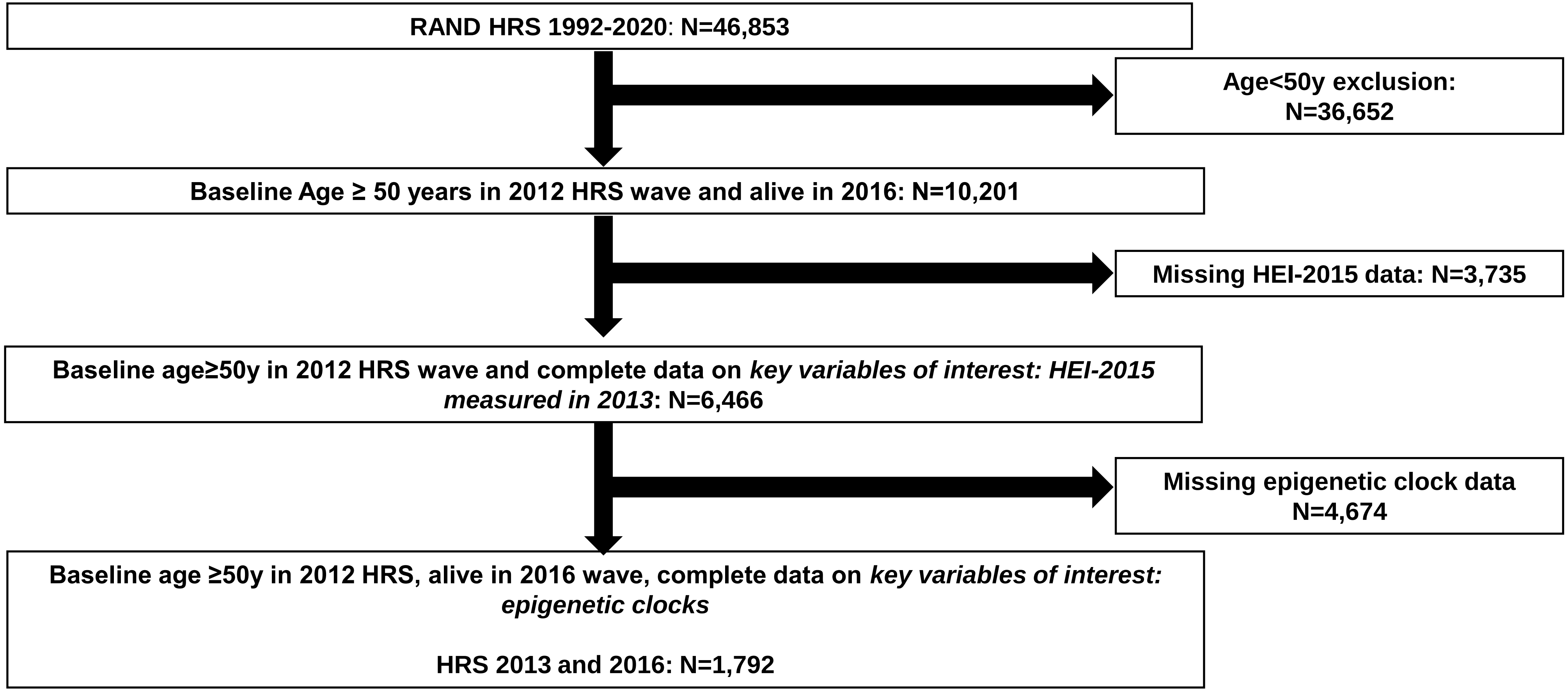

Abbreviations: HRS=Health and Retirement Study; NHANES=National Health and Nutrition Examination Surveys.
