# Supplementary material for: Healthy Eating Index, Epigenetic Age Acceleration and Mortality Risk in US Adults
Source: Aging Cell. 2026 May 5;25(5):e70504. doi: 10.1111/acel.70504 (PMC13143866; doi:10.1111/acel.70504)
Supplement: Supplementary file 2 — FIGURE S2: ABN findings using discrete time hazards models, for 1–3 parents/child limits. [file ACEL-25-e70504-s008.pdf]

FIGURE S2. ABN findings using discrete time hazards models, for 1-3 parents/child limits

(A) NHANES 1999-2002, follow-up till 2019

1 parent/child

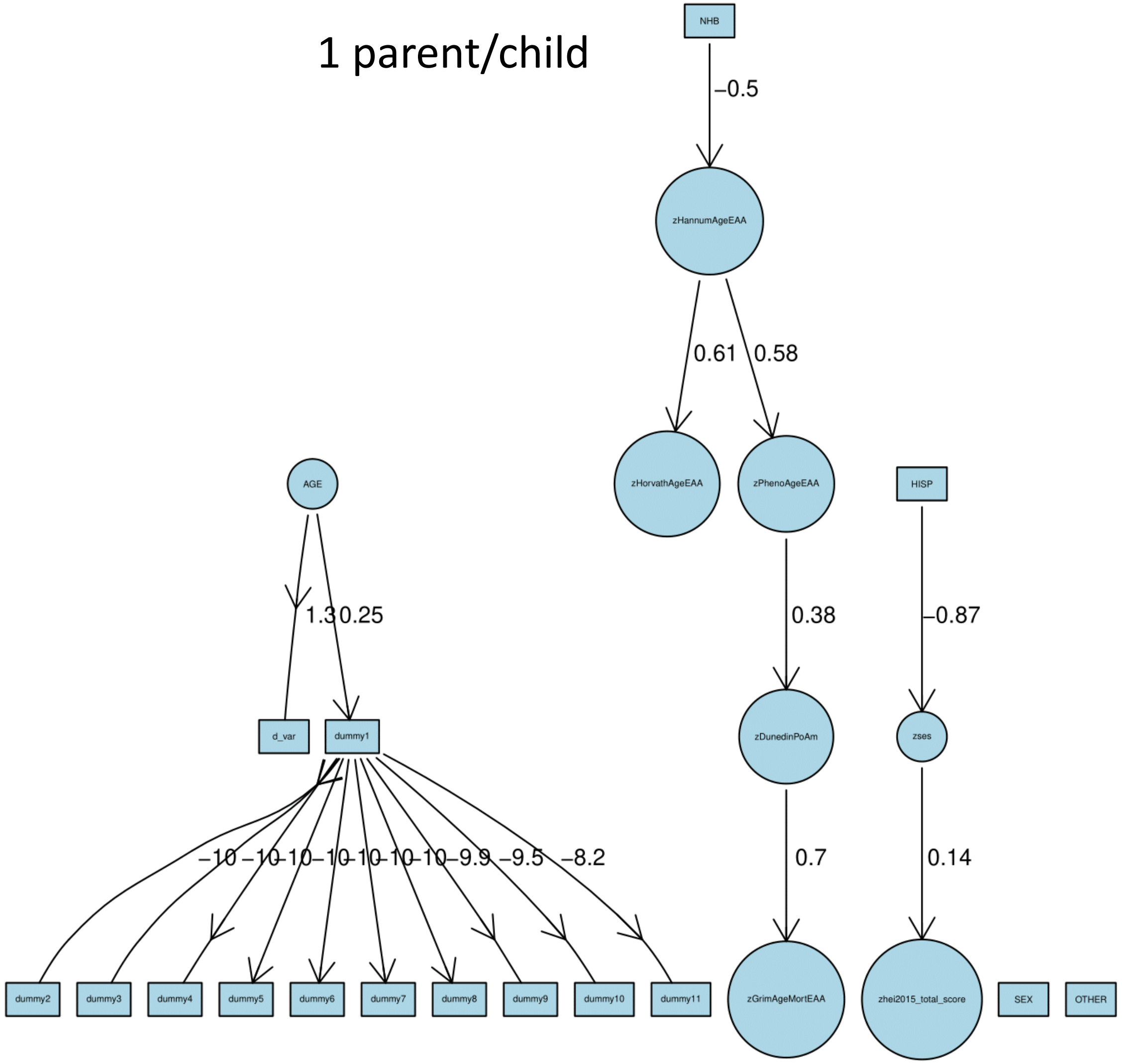

2 parents/child

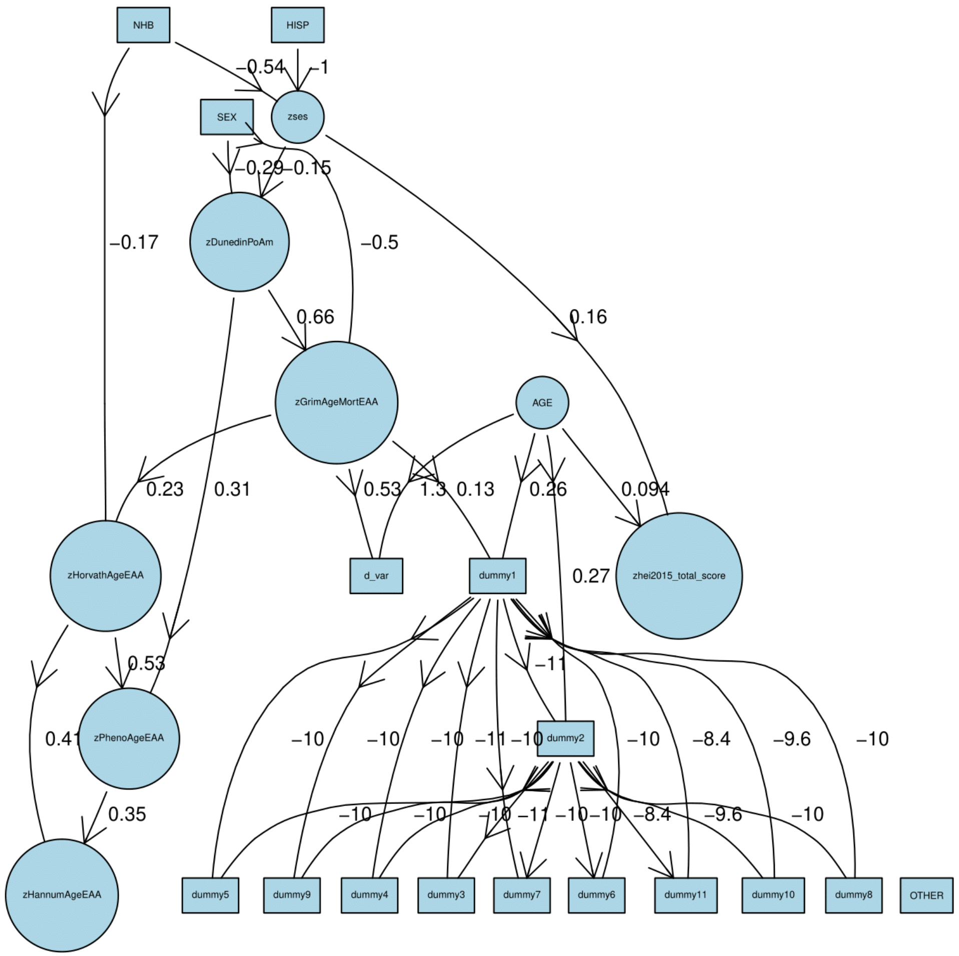

NHANES 1999-2019

Model fit for 1-3 parents/child

3 parents/child solution

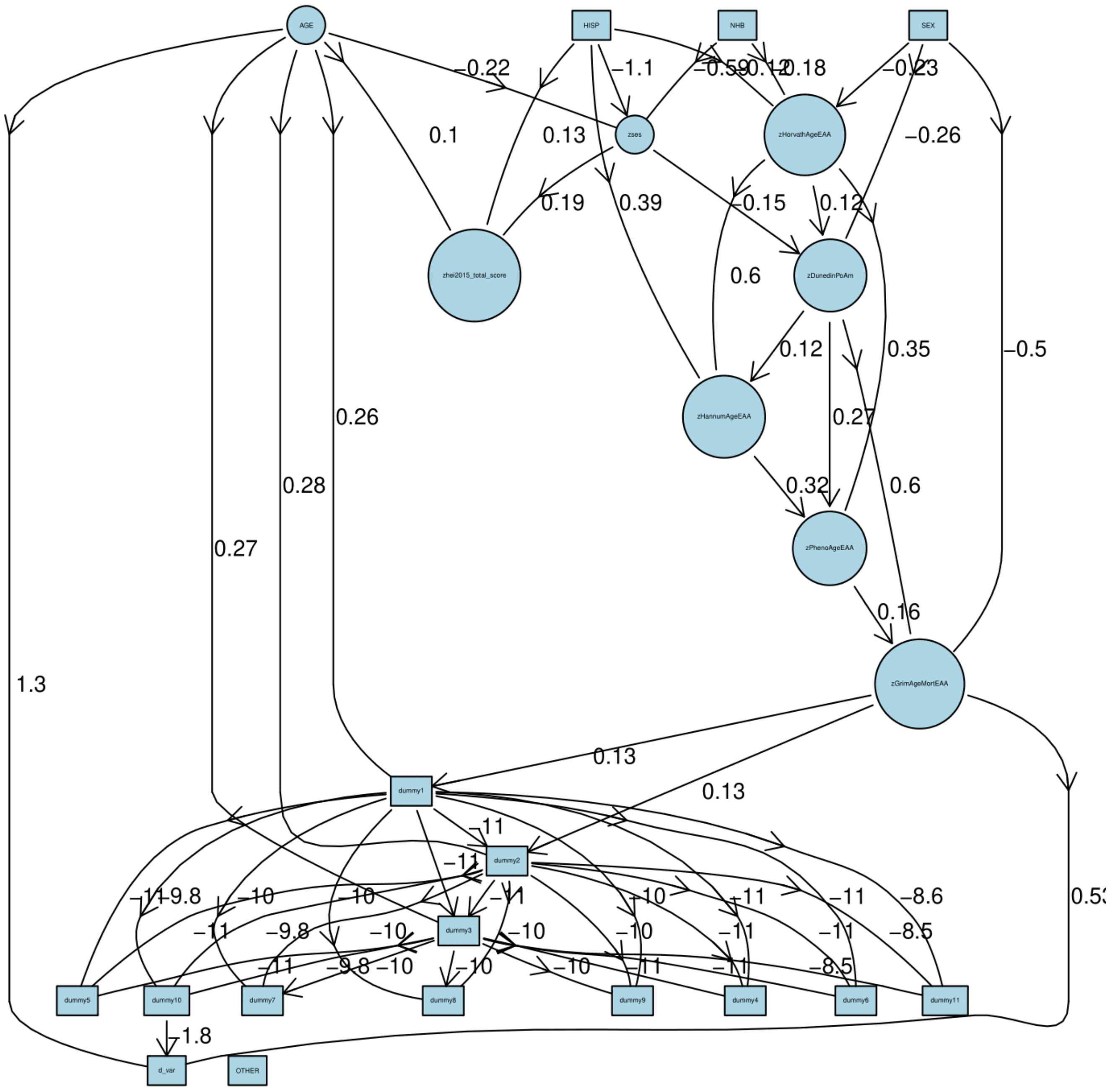

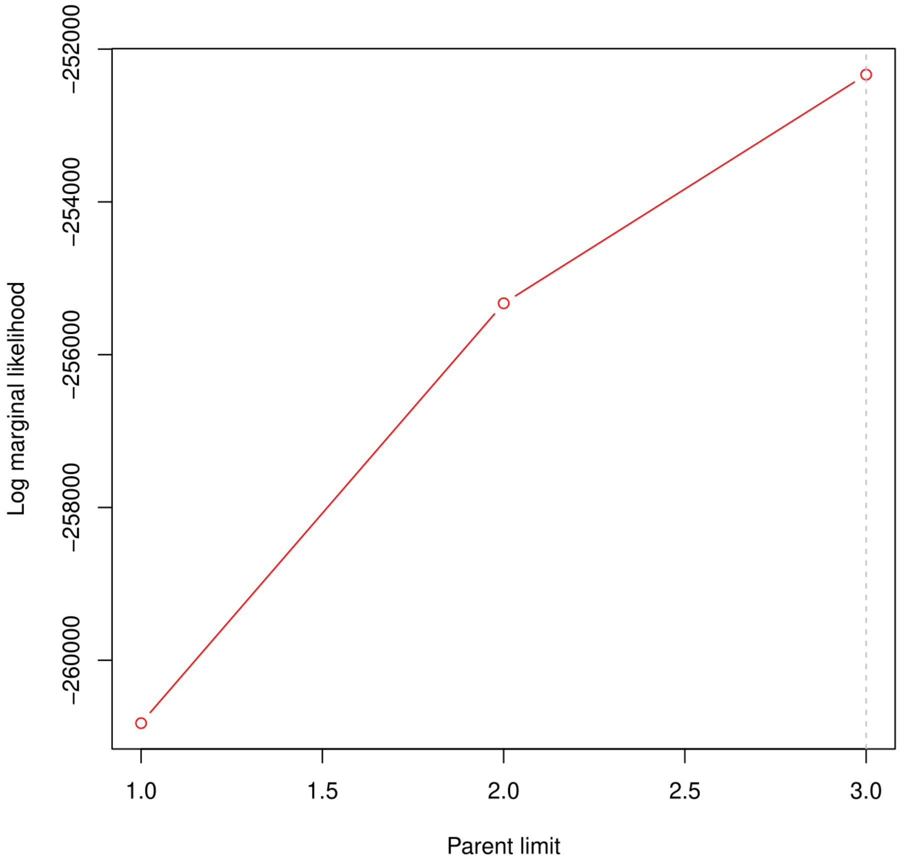

(B) HRS 2013 (HEI-2015) and 2016 (epigenetic clocks) follow-up till 2022

1 parent/child

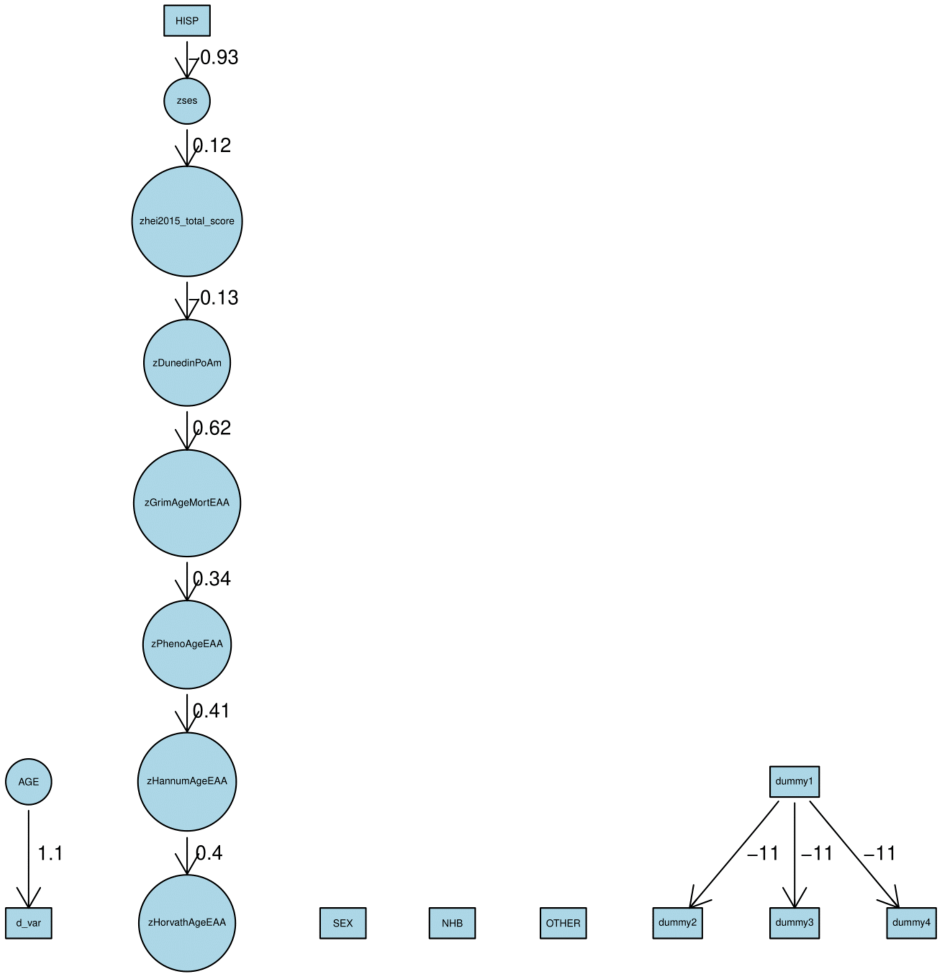

2 parents/child

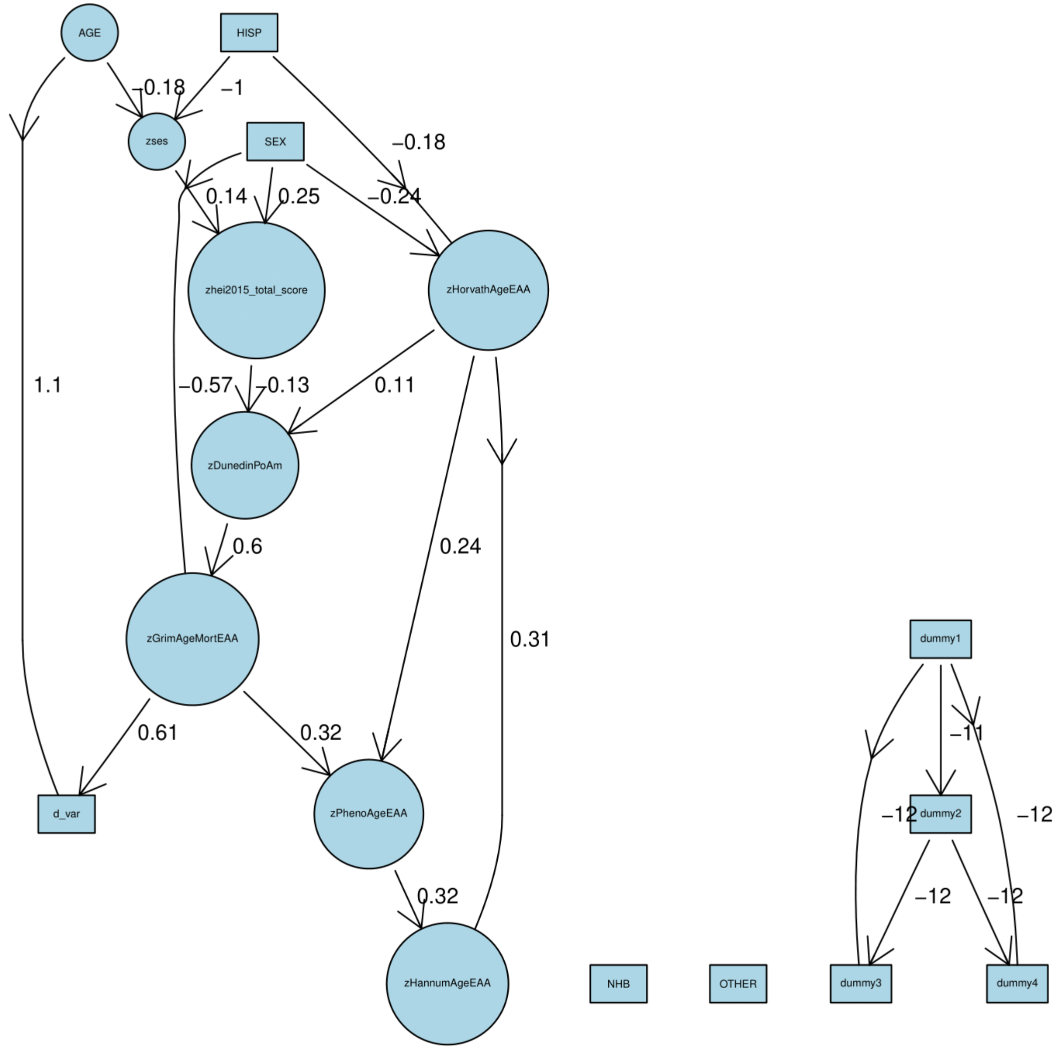

HRS 2016-2022

3 parents/child solution

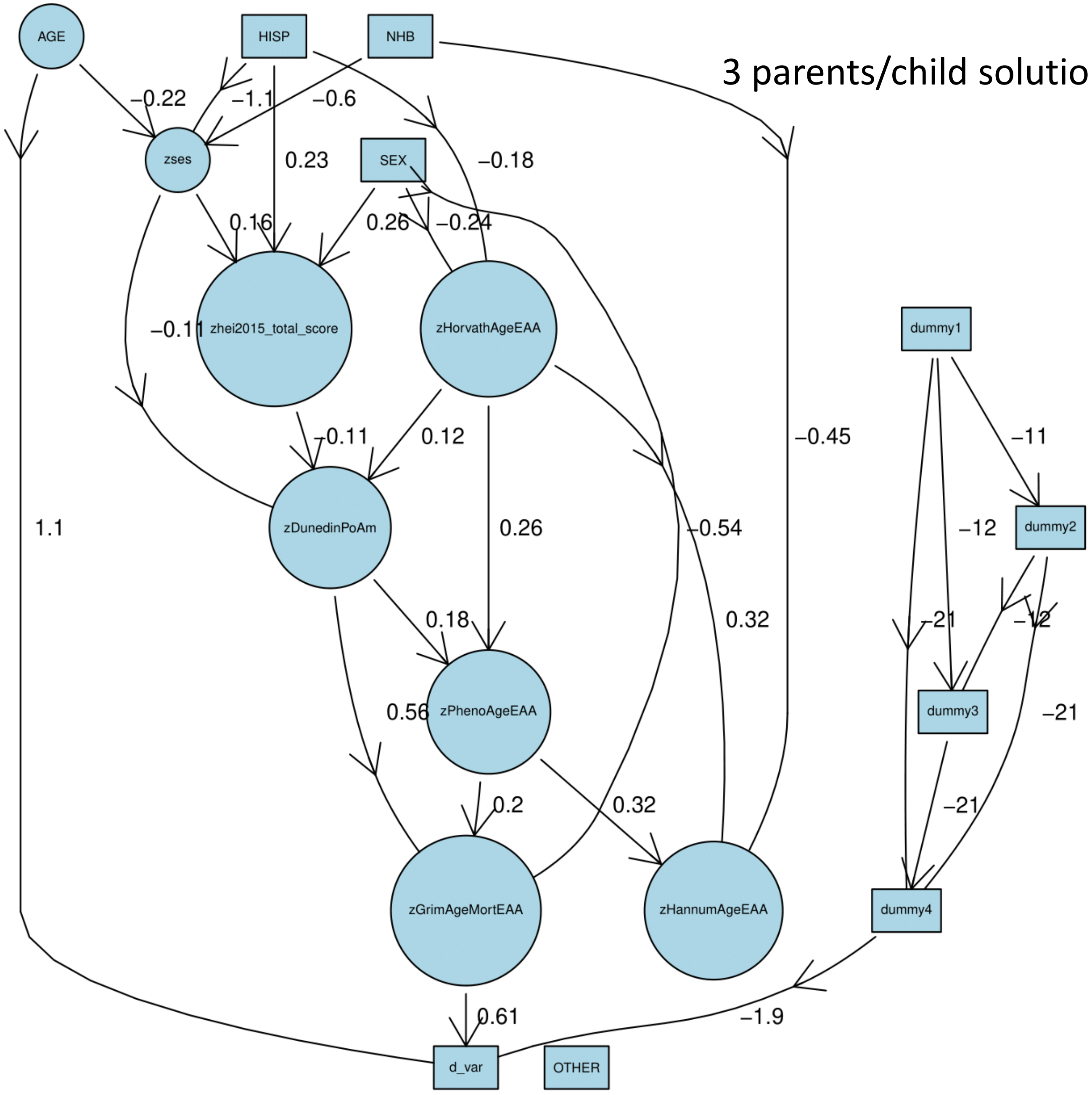

Model fit for 1-3 parents/child

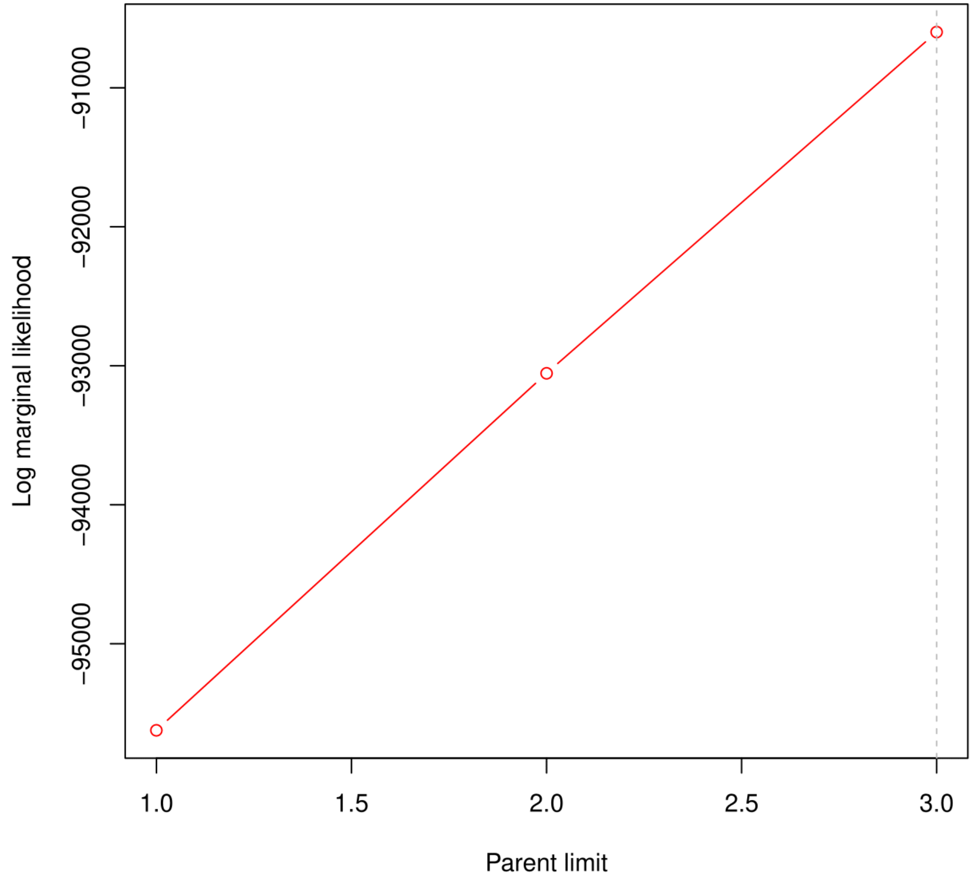

Notes: Details for R code used for this analysis described in **Appendix V** and provided on github. This code provides a comprehensive pipeline for conducting ABN analysis, including installation, data preprocessing, constraint specification, model fitting, and iterative optimization. It involves installing R versions 4.4 or higher, data preparation, data wrangling, defining variable groups, setting constraints, optimizing across parent limits, building the additive Bayesian network, and generating visual representations. The optimal number of parents of a child is determined based on levelling off the log marginal likelihood and desired complexity between key variables. Unweighted sample sizes were n=2,158 for NHANES and n=1,752 for HRS.

Abbreviations: DunedinPoAm=Dunedin Pace of Aging DNA methylation clock; GrimAgeEAA=Grim DNA methylation Epigenetic Age Acceleration; HannumAgeEAA=Hannum DNA methylation Age, Epigenetic Age Acceleration; HEI-2015=Healthy Eating Index, 2015; HorvathAgeEAA=Horvath DNA methyalation Age, Epigenetic Age Acceleration; HRS=Health and Retirement Study; NHANES=National Health and Nutrition Examination Surveys; PhenoAgeEAA=Pheno DNA methylation Age Epigenetic Age Acceleration; SES=Socio-economic Status; z=standardized z-score.
