# Supplementary material for: Healthy Eating Index, Epigenetic Age Acceleration and Mortality Risk in US Adults
Source: Aging Cell. 2026 May 5;25(5):e70504. doi: 10.1111/acel.70504 (PMC13143866; doi:10.1111/acel.70504)

Figure S3. Combined heatmaps of four-way decomposition models across primary and sensitivity analyses.

This figure presents four heatmaps summarizing four-way decomposition components for the Diet quality–epigenetic aging–mortality pathway across NHANES and HRS. Panels include: (A) primary analysis ; (B) primary analysis additionally adjusted for leukocyte composition (WBC) ; (C) reverse causation model (epigenetic aging → diet quality → mortality) ; and (D) reverse causation model with additional WBC adjustment .

Rows represent epigenetic clocks (EPICLOCK), and columns represent four-way decomposition parameters, including the controlled direct effect (CDE), pure indirect effect (PIE), mediated interaction (INTmed), reference interaction (INTref), and total effect (TE), as well as corresponding proportion parameters (p_ and op_).

Color gradients indicate the magnitude and direction of standardized coefficients (blue = negative; red = positive for main effects; green–purple scale for proportion parameters). Values within cells denote coefficients rounded to three decimals; asterisks indicate statistical significance ($P < 0.05$).

Comparative visualization allows assessment of robustness to leukocyte adjustment and evaluation of directional consistency between primary and reverse causation frameworks.

(A) Primary analysis: Diet quality → Epigenetic aging → Mortality

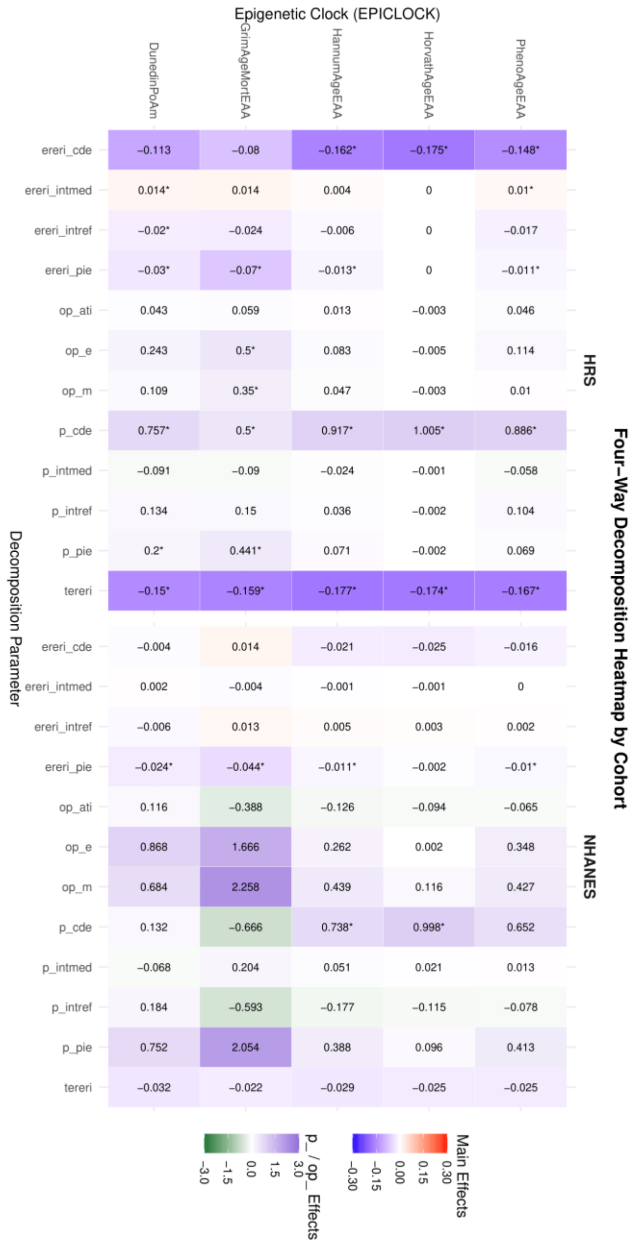

(B) Sensitivity analysis: Diet quality → Epigenetic aging → Mortality: + WBC adjustment

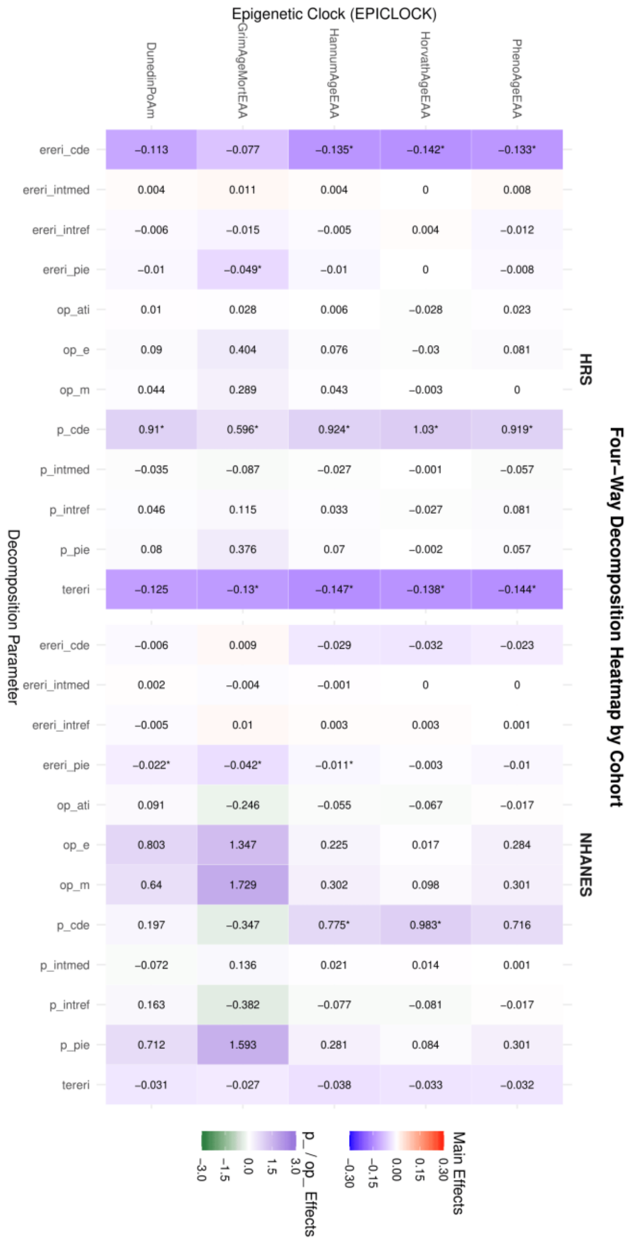

(C) Sensitivity analysis: Epigenetic aging →Diet quality→ Mortality

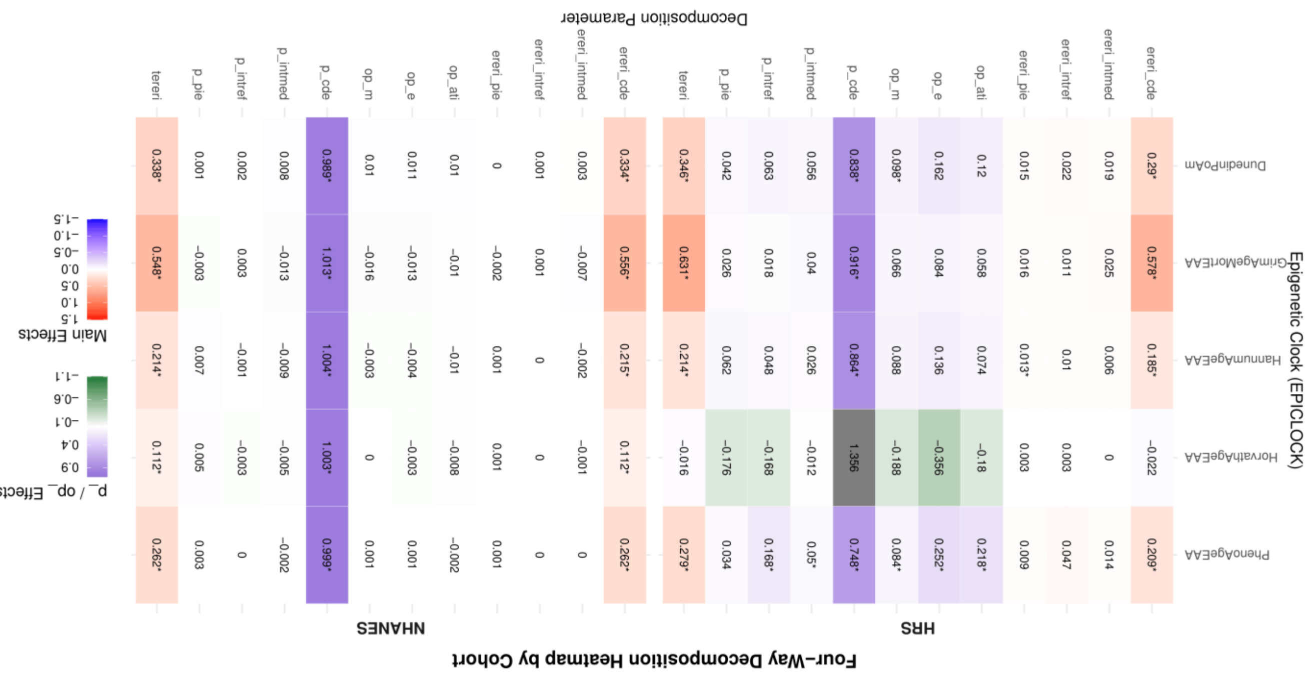

(D) Sensitivity analysis: Epigenetic aging →Diet quality→ Mortality: + WBC adjustment

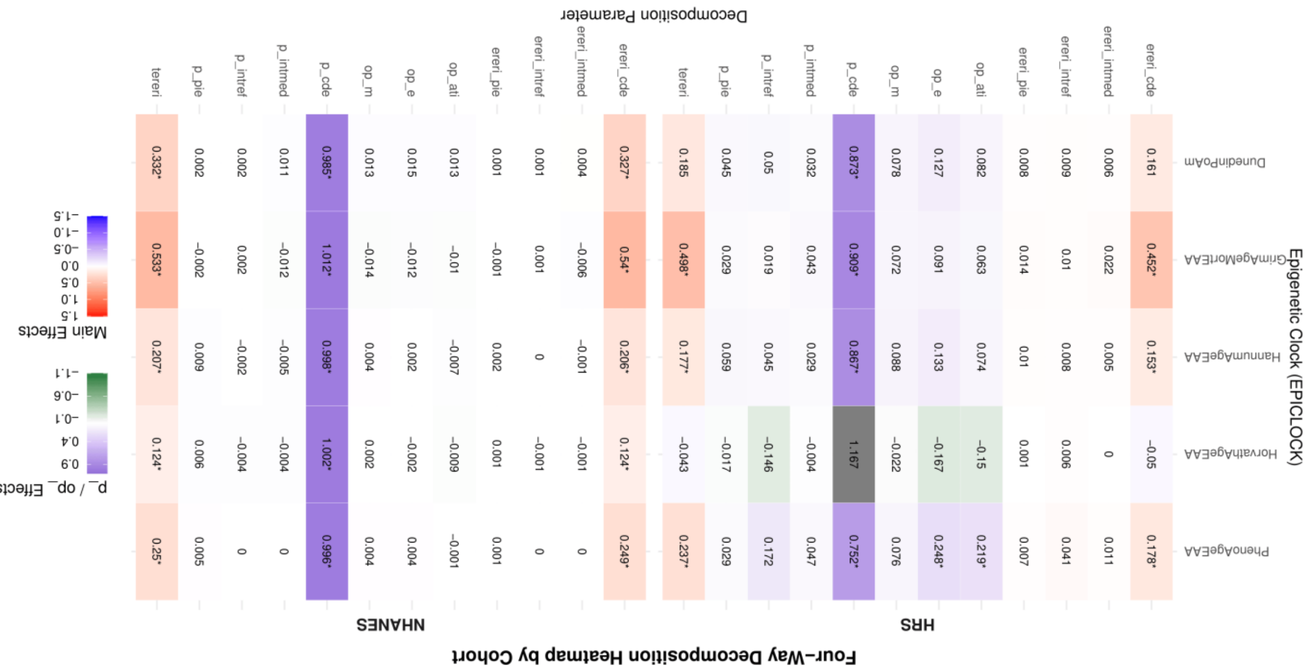

Supplement: Supplementary file 3 — FIGURE S3: Combined heatmaps of four‐way decomposition models across primary and sensitivity analyses. [file ACEL-25-e70504-s005.pdf]
