# Supplementary material for: Healthy Eating Index, Epigenetic Age Acceleration and Mortality Risk in US Adults
Source: Aging Cell. 2026 May 5;25(5):e70504. doi: 10.1111/acel.70504 (PMC13143866; doi:10.1111/acel.70504)
Supplement: Supplementary file 4 — Appendices: Comparison of Included vs. Excluded Participants and Selection Model for Analytical Samples (NHANES 1999–2002 and HRS 2013–2016) Panel A. Included vs. Excluded. [file ACEL-25-e70504-s002.pdf]

SUPPLEMENTARY MATERIALS

APPENDIX I. DATABASES AND DETAILED STUDY DESIGN DOCUMENTATION

1) NHANES:

The CDC website for the National Health and Nutrition Examination Survey (NHANES) offers detailed guidance for researchers on data interpretation. This document includes survey methodology, sample design, estimation approaches, and analytical strategies. They are often updated to reflect changes in survey design and incorporate new statistical techniques. The primary components of the NHANES analytic standards include Plan and Operations Reports, Sample Design Documentation, Estimation and Weighting Procedures, and Analytic Standards. The "National Health and Nutrition Examination Survey: Analytic Guidelines, 1999-2010" provides updated directives for data analysis from those survey periods. The "NHANES Analytic Guidance and Brief Overview for the 2017-March 2020 Pre-pandemic Data Files" clarifies the challenges in data collecting faced during the COVID-19 pandemic and provides instructions for integrating data from several cycles to obtain nationally representative estimates.
The current investigation utilized solely demographic files from the 1999-2000 and 2001-2002 cycles, which were integrated with additional excess serum data about epigenetic clocks. The demographic data encompasses all age groups from 0 to 85 years; however, this study exclusively included individuals aged 50 years and older to align with the age range of DNAm data and epigenetic clocks. A two-cycle weighting technique was implemented in the majority of analyses with four-year sample weights modified according to the availability of epigenetic data.

Source: https://wwwn.cdc.gov/nchs/nhanes/analyticguidelines.aspx

2) HRS:

The Health and Retirement Study (HRS) provides comprehensive documentation regarding its survey design and methods. The data collection path table offers a historical overview of HRS data collecting initiatives, featuring links to comprehensive information regarding each data output. The longitudinal cohort sample design demonstrates the aggregation of HRS samples across time, emphasizing distinct birth cohorts. The study currently employs a steady-state approach, renewing the sample every six years with younger cohorts. Tables present comprehensive data on sample sizes and interview response rates for each survey year of the core biennial survey, organized by overall panel, race/ethnicity, and cohort. Weight information is supplied for impartial national estimates, while resources like "An Elementary Cookbook of Data Management using HRS Data with SPSS, SAS, and Stata Examples" offer practical instruction on organizing and interpreting HRS data across many statistical software platforms. Technical studies provide comprehensive details on the HRS sample design, encompassing approaches such as unfolding brackets to mitigate item nonresponse in economic surveys. The discussion encompasses imputations, outlining the methodologies utilized across different survey waves. Administrative material offers insights into Institutional Review Board (IRB) considerations and other relevant administrative factors related to the HRS. These tools combined provide a comprehensive overview of the HRS's survey design and methodology, assisting researchers in properly employing the data for their studies. Alongside the Core data that can be associated with the tracker files, the RAND HRS data product is available for download at: https://hrsdata.isr.umich.edu/data-products/rand?_gl=1*65fx5r*_ga*. The project utilizes the newest version issued in May 2024, specifically the 2020 HRS RAND FAT FILE, V1.A. The tracker file utilized for the current analysis is the most recent version, issued in November 2024, and extends to early 2022 in follow-up. The URL is: https://hrsdata.isr.umich.edu/data-products/cross-wave-tracker-file?_gl=1*1njsolk*_ga*MTA3MTg2OTA4NS4xNzMxNjIwNDk3*_ga_FF28MW3MW2*MTczNDE4O

TM1MS41LjEuMTczNDE5MDE0OS4wLjAuMA. The Health and Retirement Study (HRS) Tracker File serves as an extensive resource for academics, including a singular record for each participant interviewed. It is revised with new data after each survey wave. The 2022 Tracker File comprises data from all cohorts participating in the 2022 data collection. This study primarily utilizes data to connect HRS participants with their date of death, hence predicting follow-up duration for conducting various survival studies.

Source: https://hrs.isr.umich.edu/documentation/survey-design

APPENDIX II. DIETARY COMPONENT AND DIET QUALITY DOCUMENTATION

1) NHANES

The NHANES 1999-2002 collected dietary data using 24-hour recalls, with a single 24-hour recall used in the present study. The data were analyzed using the Automated Multiple-Pass Method (AMPM) and the USDA Food and Nutrient Database for Dietary Studies (FNDDS). These data were used to assess macronutrient and micronutrient intakes, food consumption patterns, and overall diet quality. The Healthy Eating Index-2015 (HEI-2015) is a measure of diet quality aligned with the 2015-2020 Dietary Guidelines for Americans. It evaluates adherence to recommended dietary patterns using 13 components, divided into adequacy and moderation categories. Each component is scored from 0 to 5 or 10, with a total possible HEI-2015 score of 100. The HEI-2015 scores were converted from NHANES data using a simple scoring algorithm.

The algorithm can be summarized as follows:

This Stata script calculates Healthy Eating Index-2015 (HEI-2015) scores using a single 24-hour dietary recall from NHANES 1999–2000 and 2001–2002 combined with Food Patterns Equivalents Database (FPED). The process involves several key steps:

1. Data Preparation & Cleaning
    - Load NHANES dietary data (HEI2015.dta).
    - Drop existing variables that might be outdated or need redefinition.
    - Generate new variables from NHANES dietary intake data by renaming relevant nutrient and food group variables.

2. Adjustments for Legume Contribution

Legumes can contribute to both protein and vegetable components of HEI-2015, so the script distributes legume intake accordingly:

- Determine total meat consumption (allmeat) including meat, poultry, fish, eggs, nuts, and soy.
- Set a meat intake threshold (mbmax) based on 2.5 oz per 1000 kcal.
- If an individual's meat intake is below the threshold (mbmax):
    - All legumes are assigned to protein intake (all2meat).
- If legume intake exceeds what is needed for protein, the excess legumes count as vegetables (meatveg condition).
- If an individual meets the meat requirement, all legumes are assigned as vegetables (all2veg condition).

3. Calculation of HEI-2015 Components
Each HEI-2015 component, excluding fatty acids, added sugar and saturated fat, is calculated based on dietary density (amount per 1000 kcal):
Adequacy Components (Higher Values Indicate Better Adherence)

1. Total Vegetables (heix1_totalveg) → Derived from legume-adjusted vegetable intake.
2. Greens & Beans (heix2_greens_and_bean) → Derived from dark green vegetables & legumes.
3. Total Fruits (heix3_totalfruit) → Total fruit intake included 100% fruit juice per 1000 kcal.
4. Whole Fruits (heix4_wholefruit) → Total fruit minus fruit juice.
5. Whole Grains (heix5_wholegrain) → Whole grain intake per 1000 kcal.
6. Dairy (heix6_totaldairy) → Total dairy intake per 1000 kcal.
7. Total Protein Foods (heix7_totprot) → Legume-adjusted total protein per 1000 kcal.
8. Seafood & Plant Protein (heix8_seaplant_prot) → Seafood and plant-based protein per 1000 kcal.
9. Fatty Acid Ratio (heix9_fattyacid) → (Monounsaturated + Polyunsaturated Fat) / Saturated Fat.

Moderation Components (Lower Values Indicate Better Adherence)

10. Sodium (heix10_sodium) → Sodium per kcal, with an ideal range (1.1–2 g).
11. Refined Grains (heix11_refinedgrain) → Scored inversely based on refined grain intake.
12. Added Sugar (heix12_addedsugar) → Percent of calories from added sugar.
13. Saturated Fat (heix13_saturatedfat) → Percent of calories from saturated fat.

Each component is capped at its maximum possible score (e.g., heix1_totalveg max = 5, heix5_wholegrain max = 10).

4. Computation of Total HEI-2015 Score

- The final HEI-2015 score (hei2015_total_score) is calculated by summing all 13 individual component scores.
- Variables are labeled appropriately for clarity.

2) HEALTH AND RETIREMENT STUDY

HRS 2013 Food Component Overview

The Health and Retirement Study (HRS) 2013 collected dietary intake data through a food frequency questionnaire (FFQ) rather than 24-hour dietary recalls. The FFQ assessed the frequency and portion sizes of various food groups consumed over a reference period (past 12 months). The main features of the HRS 2013 dietary data include:

- Self-reported intake of key food groups (e.g., fruits, vegetables, dairy, grains, protein sources, fats, and sugars).
- Approximate nutrient composition derived from standard reference databases.
- Limited detail on portion sizes compared to NHANES, requiring conversion to standardized servings for HEI-2015 computation.

Computing HEI-2015 Using HRS 2013 Data

Since **HRS uses an FFQ**, computing the **Healthy Eating Index-2015 (HEI-2015)** involves the following steps:

1. Mapping HRS Food Groups to HEI-2015 Components

Each HEI-2015 component requires **food group equivalents per 1000 kcal**. Given HRS data do not provide direct nutrient density measures, food groups must be **converted into standardized servings and energy-adjusted**:

HEI-2015 Component	HRS 2013 Food Groups Used
Total Vegetables	Frequency of dark green, orange, starchy, and other vegetables

HEI-2015 Component	HRS 2013 Food Groups Used
Greens & Beans	Dark green vegetables & legumes
Total Fruits	Frequency of all fruits, including juice
Whole Fruits	Whole fruits excluding juice
Whole Grains	Whole grain foods
Dairy	Milk, yogurt, cheese intake
Total Protein Foods	Meat, poultry, eggs, fish, nuts, soy, legumes
Seafood & Plant Proteins	Fish, nuts, soy, legumes
Fatty Acid Ratio	Derived from reported intake of fats
Sodium	Estimated based on reported processed and salty foods
Refined Grains	White bread, pasta, rice, processed grains
Added Sugars	Sugar-sweetened beverages, sweets
Saturated Fat	High-fat dairy, butter, fried foods

2. Estimating Energy Density (per 1000 kcal)

- HRS does not provide total energy intake directly like NHANES.
- Researchers typically use an estimation model based on sex, age, weight, and reported intake patterns to approximate kcal intake.
- Once estimated kcal intake is available, food groups are expressed as servings per 1000 kcal to align with HEI-2015 scoring.

3. Computing HEI-2015 Scores

HEI-2015 Component	Scoring Criteria
Total Vegetables	5 points (1.1 cups/1000 kcal)
Greens & Beans	5 points (0.2 cups/1000 kcal)
Total Fruits	5 points (0.8 cups/1000 kcal)
Whole Fruits	5 points (0.4 cups/1000 kcal)
Whole Grains	10 points (1.5 oz/1000 kcal)
Dairy	10 points (1.3 cups/1000 kcal)
Total Protein Foods	5 points (2.5 oz/1000 kcal)
Seafood & Plant Proteins	5 points (0.8 oz/1000 kcal)
Fatty Acid Ratio	10 points (monounsaturated + polyunsaturated fat to saturated fat ratio: 2.5 or higher)
Sodium	10 points (≤1.1 g/1000 kcal)
Refined Grains	10 points (≤1.8 oz/1000 kcal)
Added Sugars	10 points (≤6.5% of kcal)
Saturated Fat	10 points (≤7% of kcal)

Each score is capped at its **maximum value**. If intake exceeds limits (e.g., too much refined grains or added sugar), the score is reduced.

4. Summing Component Scores for Total HEI-2015

- The HEI-2015 total score is obtained by summing all 13 components.
- The final HEI-2015 score ranges from 0 to 100, with higher scores indicating greater adherence to dietary guidelines.

Both NHANES and HRS Stata codes will provided for further details on the github repository.

Source: https://hrsdata.isr.umich.edu/data-products/2013-health-care-and-nutrition-study-hcns

APPENDIX III. EPIGENETIC CLOCK DOCUMENATION

Health and Retirement Study (HRS)

Epigenetic data were obtained from a subsample of 4,018 HRS participants, with a high success rate—over 97%—in DNA methylation profiling. Methylation was measured using the Illumina Infinium MethylationEPIC BeadChip array. To ensure comparability with NHANES and other large-scale studies, five established epigenetic clocks were selected: Horvath, Hannum, Levine PhenoAge, GrimAge, and Dunedin Pace of Aging (DunedinPACE). For the first four clocks, epigenetic age acceleration (EAA) was calculated by regressing epigenetic age on chronological age and using the residuals as indicators of biological age not explained by calendar age. These residuals could be negative or positive, reflecting biological age either below or above expected for chronological age. Because DunedinPACE inherently measures the rate of aging, no transformation was applied. All five metrics were standardized (z-scored) after excluding outliers from the final analytic sample.
Source: https://hrsdata.isr.umich.edu/data-products/epigenetic-clocks and (Beydoun et al., 2022)

National Health and Nutrition Examination Survey (NHANES)

Comprehensive documentation of NHANES DNA methylation and epigenetic biomarker data is publicly available (CDC DNAm Resource). Methylation was measured using the Illumina MethylationEPIC BeadChip for adults aged 50 years and older, with rigorous bioinformatics preprocessing, normalization, and quality control. This includes exclusion of outliers and mismatched samples, as well as estimation of blood cell-type proportions. Consistent with the approach used in HRS, we selected the Horvath, Hannum, PhenoAge, GrimAge, and DunedinPACE clocks for analysis. EAA metrics for the first four clocks were derived using the residual method, while DunedinPACE was analyzed without modification as a direct measure of aging rate.

APPENDIX IV. DISCRETE TIME HAZARD MODEL:

Discrete time hazard models are statistical techniques employed to examine time-to-event data when the time variable is transformed into discrete intervals, such as years, months, or days. These models are commonly utilized in the social sciences, public health, and educational research. The primary characteristics of discrete time hazard models encompass temporal disclosure, probabilistic modeling, adaptable covariates, and the binary logistic regression framework. These strengths allows it be adaptable to additive Bayesian networks, which can only incorporate certain distributions for variables, such as Gaussian, binomial, and Poisson distributions for the variables within the model (See **Appendix V**).

The implementation of discrete time hazard modeling involves the formation of time periods, specification of baseline hazard, incorporation of covariates, model estimate, and interpretation. The pros for utilizing discrete time hazard models include the good management for tied event times, integration of time-varying covariates, and straightforward application utilizing logistic regression. The limitations of discrete time hazard models encompass the discretization of continuous time data, the loss of information when time intervals are extensive, and the presumption of uniform risk within each time interval. Common tools for executing discrete temporal hazard models are available in R, Stata, and SAS.

Discrete time hazard models are logistic regression models utilized on data formatted in person-period, incorporating many dummy variables to replicate a hazard function.

$$\log\left(\frac{h_t}{1-h_t}\right) = \beta_0 + \sum_{i=1}^{p} \beta_i X_i$$

Sources: (Kvamme & Borgan, 2021)

APPENDIX V. ADDITIVE BAYESIAN NETWORKS:

A) Theoretical framework

Additive Bayesian networks (ABNs) are probabilistic graphical models that utilize a directed acyclic graph (DAG) to depict conditional relationships among variables. They provide advantages including multivariate modeling, causal interpretation, and flexibility in handling different kinds of data and distributions. ABNs are constructed by estimating local distributions for each node, utilizing linear regression for continuous data and logistic regression for binary variables. Bayes' Theorem is fundamental to ABN, as it seeks to deduce the posterior distribution of model parameters from the data. The software computes the posterior distribution by integrating the likelihood derived from the data with the specified priors. A scoring function determines the optimal configuration for the ABN, while the Bayesian Information Criterion (BIC) is utilized to balance goodness-of-fit with model complexity. ABN is extensively utilized in fields such as epidemiology, genetics, and social sciences to analyze multivariate correlations and infer causal relationships.

Sources: (Lewis & Ward, 2013; Scutari, 2022)

The following set of equations are used in this method:

(Eq. 1.1) *Linear regression*: $Y = \beta_0 + \sum_{i=1}^{k} \beta_i X_i + \varepsilon$

(Eq. 1.2) *Logistic regression*: $logit\big(P(Y = 1|X_1, \dots, X_k)\big) = \beta_0 + \sum_{i=1}^{k} \beta_i X_i$

(Eq. 1.3) *Likelihood Function*: $\boldsymbol{L(\theta|D) = \prod_{i=1}^{n} P(X_i \mid Parents(X_i), \theta_i)}$

(Eq. 1.4) *Bayesian Posterior*: $\boldsymbol{P(\theta|D) = \frac{P(D|\theta)P(\theta)}{P(D)}}$

(Eq. 3.5) *BIC for Model Selection*: $\boldsymbol{BIC = -2log\big(L(\theta|D)\big) + p \times log(n)}$

B) Additive Bayesian Network workflow

The R code, which is provided on github and applied to both the NHANES and HRS datasets, offers a thorough pipeline for doing ABN analysis, encompassing installation, data preprocessing, constraint formulation, model fitting, and iterative optimization.

This study established the best number of parents for the child by analyzing the stabilization of the log marginal likelihood and the requisite complexity among key variables. Owing to the substantial computing demands of this method and the considerable sample size (e.g., >18,000 person-period sample for NHANES 1999-2019), a maximum of 3 parents/child were included. Consequently, two parents per child were only deemed relevant if there was a significant stabilization of the marginal likelihood between two and three parents per child.

Source: https://r-bayesian-networks.org/

APPENDIX VI. GENERALIZED STRUCTURAL EQUATIONS MODELS

Stata's Generalized Structural Equation Modeling (gsem) is a flexible tool that estimates linear and non-linear relationships among variables, accommodating different dependent variables and random effects. It uses Maximum Likelihood or Quasi-ML methods, and Goodness-of-Fit statistics and tests to evaluate model alignment with data. Users can perform linear predictions, estimate residuals, and calculate linear and non-linear combinations of factors.

Probability density function for Weibull distribution

$$f(t;\delta,k)=\frac{k}{\delta}(\frac{t}{\delta})^{k-1}e^{-(\frac{t}{\delta})^{k}}, t\geq 0$$

Where:

- *t is the time or random variable of interst.*
- *δ>0 is the scale parameter*
- *k>0 is the shape parameter*

Cumulative distribution function (CDF) and survival function (complement of CDF) for Weibull distribution

$$F(t;\delta,k)=1-\ e^{-(\frac{t}{\delta})^{k}}, t\geq 0$$

$$S(t;\delta,k)=e^{-(\frac{t}{\delta})^{k}}, t\geq 0$$

Hazard function for Weibull distribution

$$h(t;\delta,k) = \frac{f(t;\delta,k)}{S(t;\delta,k)} = \frac{k}{\delta}\left(\frac{t}{\delta}\right)^{k-1}, t \geq 0$$

Source: https://www.stata.com/manuals/semgsem.pdf

APPENDIX VII. FOUR-WAY DECOMPOSTION MODEL

A7.1. Notation

Let

- A= exposure (HEI-2015; contrast $a_1$vs a_0)
- M= mediator (EAA metric)
- T= survival time
- M_a= mediator under $A = a$
- $Y_{a,m}(t)$= event status by time $t$if $A = a$, $M = m$
- m^*= fixed mediator value (m(#) option)

The total effect (TE) on the risk ratio (RR) scale is

$$RR_{TE} = \frac{Pr(Y_{a_1,M_{a_1}} = 1)}{Pr(Y_{a_0,M_{a_0}} = 1)}.$$

For Cox models, med4way reports effects on the excess relative risk scale:

$$TERERI = RR_{TE} - 1.$$

A7.2. Four Components

On the ratio scale:

Controlled Direct Effect (CDE):

$$RR_{CDE}(m^*) = \frac{Pr(Y_{a_1,m^*} = 1)}{Pr(Y_{a_0,m^*} = 1)}$$

Pure Indirect Effect (PIE):

$$RR_{PIE} = \frac{Pr(Y_{a_0,M_{a_1}} = 1)}{Pr(Y_{a_0,M_{a_0}} = 1)}$$

Reference Interaction (INTref) and Mediated Interaction (INTmed) capture exposure–mediator interaction components (without and with mediation, respectively).

On the excess scale:

$$TERERI = ERERI_{CDE} + ERERI_{INTref} + ERERI_{INTmed} + ERERI_{PIE},$$

where each $ERERI = RR - 1$.

A7.3. Model Specification

Mediator model (linear):

$$M = \alpha_0 + \alpha_A A + \alpha_C C + \varepsilon$$

Outcome model (Cox):

$$h(t \mid A, M, C) = h_0(t)\exp\left(\beta_A A + \beta_M M + \beta_{AM} A \times M + \beta_C C\right)$$

Covariates C include demographics and SES.

A7.4. med4way, fulloutput — Reported Quantities (Cox)

Core quantities (always reported; excess scale):

- tereri = total excess effect
- ereri_cde = CDE component
- ereri_intref = reference interaction
- ereri_intmed = mediated interaction
- ereri_pie = pure indirect effect

With fulloutput, additionally:

- terira = total effect on RR scale (i.e., RR_{TE})

Proportions of total excess effect:

- p_cde = ERERI $_{CDE}$/TERERI
- p_intref = ERERI $_{INTref}$/TERERI
- p_intmed = ERERI $_{INTmed}$/TERERI
- p_pie = ERERI $_{PIE}$/TERERI

Overall summaries:

- op_m = overall proportion mediated
$$(ERERI_{PIE} + ERERI_{INTmed})/TERERI$$
- op_ati = overall proportion attributable to interaction
$$(ERERI_{INTref} + ERERI_{INTmed})/TERERI$$
- op_e = overall proportion eliminated if M fixed at m^*

For Cox models, these quantities rely on the rare-outcome approximation, interpreting hazard ratios as approximations to risk ratios.

A.7.5 White blood cell composition metrics

For harmonized analyses of leukocyte composition, comparable complete blood count (CBC) measures are available from NHANES 1999–2002 and the HRS 2016 Venous Blood Study (VBS). NHANES documentation can be accessed at:
1999–2000: https://wwwn.cdc.gov/Nchs/Data/Nhanes/Public/1999/DataFiles/LAB25.htm
2001–2002: https://wwwn.cdc.gov/Nchs/Data/Nhanes/Public/2001/DataFiles/L25_B.htm
In NHANES 1999–2002, venous blood samples were obtained in Mobile Examination Centers and analyzed using standardized automated hematology analyzers under rigorous quality-control procedures. The CBC panel includes total white blood cell (WBC) count and a five-part differential reported both as percentages and absolute counts. The differential comprises neutrophils, lymphocytes, monocytes, eosinophils, and basophils, each expressed as a percentage of total WBC and as absolute counts (cells $\times 10^3/\mu L$). For harmonization purposes, total WBC count and the five differential percentages are the primary variables of interest, as they characterize circulating immune cell composition independently of erythrocyte and platelet indices. These indicators are commonly used to account for immune cell

heterogeneity in studies of inflammation, epigenetic aging, transcriptomics, and related molecular outcomes.

The HRS 2016 Venous Blood Study provides directly comparable venous-based CBC measures collected by trained phlebotomists during in-home visits and processed in certified laboratories using automated hematology platforms. Documentation is available at: https://hrsdata.isr.umich.edu/documentation (see 2016 Venous Blood Study files). Similar to NHANES, the HRS VBS includes total WBC count and a five-part differential, reported as both percentages and absolute counts for neutrophils, lymphocytes, monocytes, eosinophils, and basophils. Because both studies rely on venous specimens and automated differential counting, leukocyte subtype percentages are methodologically comparable across cohorts.

For harmonized modeling, the shared variables include total WBC count and the percentage of neutrophils, lymphocytes, monocytes, eosinophils, and basophils. Differential percentages provide a standardized representation of leukocyte composition suitable for covariate adjustment, helping to minimize confounding arising from variation in circulating immune cell mixtures. Using percentages rather than absolute counts also improves cross-cohort comparability when minor differences in laboratory platforms or calibration exist.

Accordingly, in sensitivity analyses conducted in both NHANES 1999–2002 and HRS 2016, WBC composition was incorporated into the four-way decomposition models by entering the common leukocyte subtype percentages as exogenous variables alongside the primary covariates. The final set of covariates included in these sensitivity models is detailed in the main Methods section.

Sources: (Discacciati, Bellavia, Lee, Mazumdar, & Valeri, 2018; VanderWeele, 2014)

APPENDIX VIII. Sensitivity analysis: Two-stage Heckman selection model

A Heckman-style two-stage selection model was implemented to assess potential bias arising from non-random inclusion into the analytic sample. First-stage probit models estimated selection probabilities using demographic predictors (age, sex, race/ethnicity). The inverse Mills ratio derived from these models was incorporated into second-stage outcome models (Cox and GSEM). Consistent with the results presented in Supplementary **Table S1**, inclusion of the inverse Mills ratio did not materially alter effect estimates, suggesting limited impact of selection bias on the primary findings.

In NHANES, included participants differed from excluded individuals by having a lower proportion of females but higher proportions of non-Hispanic Black and Hispanic participants, with all differences statistically significant except for other race/ethnicity. In HRS, included participants were more likely to be female and Hispanic but less likely to be non-Hispanic Black, with modest but statistically significant differences, while other race/ethnicity showed no difference between groups.

Incorporation of the inverse Mills ratio into GSEM models provided little evidence that selection bias materially influenced the primary associations of interest. The IMR was not associated with mortality risk, diet quality, socioeconomic status, or most epigenetic aging markers. A consistent association was observed only for GrimAgeEAA, suggesting some degree of selection related to this specific biomarker. However, because this did not translate into changes in the mortality pathway or overall model structure, the primary findings appear robust to selection bias. The full GSEM models with inverse Mills ratio incorporated into the equations are presented in **Table S2**.

Table S1. Comparison of Included vs. Excluded Participants and Selection Model for Analytical Samples (NHANES 1999–2002 and HRS 2013–2016) **Panel A. Included vs. Excluded**

Panel A. Included vs. Excluded Participants

Variable	NHANES Included (n = 2,158)	NHANES Excluded (n = 8,133)	P-value	HRS Included (n = 1,792)	HRS Excluded (n = 44,297)	P-value
Age, mean (SE)	—	—	—	—	—	—
Female, %	49.21	54.40	<0.001	59.32	55.49	0.001
NHB, %	20.48	18.20	0.015	14.79	16.94	0.017
Hispanic, %	34.48	27.03	<0.001	12.44	10.86	0.035
Other race/ethnicity, %	3.24	3.50	0.556	3.12	3.25	0.764

Panel B. Probit Selection Model for Inclusion in Analytical Sample

Predictor	NHANES Coefficient (SE)	P-value	HRS Coefficient (SE)	P-value
Age	0.042 (0.001)	<0.001	0.015 (0.001)	<0.001
Female	−0.117 (0.032)	<0.001	0.031 (0.026)	0.226
NHB	0.487 (0.044)	<0.001	−0.217 (0.035)	<0.001
Hispanic	0.642 (0.039)	<0.001	−0.149 (0.038)	<0.001
Other race	0.462 (0.091)	<0.001	−0.211 (0.068)	0.002
Constant	−3.415 (0.071)	<0.001	−2.285 (0.085)	<0.001

NHANES: Pseudo $R^2 = 0.225$; LR $\chi^2 = 2378.99$, $P < 0.001$
HRS: Pseudo $R^2 = 0.023$; LR $\chi^2 = 273.12$, $P < 0.001$

APPENDIX IX. Sensitivity analysis: Adjustment of HRS four-way decomposition models for lifestyle factors (smoking, physical activity, alcohol use, and total energy intake)

This sensitivity analysis was conducted to evaluate whether the main mediation findings were robust to additional adjustment for lifestyle behaviors and dietary intake. To do this, information on smoking, alcohol use, and physical activity from the 2016 wave of the Health and Retirement Study (HRS) was added, along with total daily energy intake (kilocalories) obtained from the dietary assessment used to compute the Healthy Eating Index (HEI) . These variables were merged at the participant level and incorporated into the analytic dataset.

Each lifestyle factor was categorized to reflect meaningful behavioral groups. Smoking status was classified as never, former, or current smoker. Alcohol use was grouped into abstinent, occasional (1–3 days per month), moderate (1–2 days per week), and frequent (≥3 days per week). Physical activity was categorized into low, moderate, and high levels based on reported frequency of moderate and vigorous activity. Energy intake was treated as a continuous measure representing total caloric consumption.

The mediation analysis used the med4way approach to examine how diet quality (HEI) relates to mortality both directly and indirectly through epigenetic aging markers. This method separates the overall association into components that reflect direct effects, indirect (mediated) effects, and their interaction. By including lifestyle behaviors and energy intake, this sensitivity analysis tested whether these factors explained or altered the observed relationships.

They key findings are provided in **supplementary datasheet 7** and summarized in the main Results section.

Supplementary References:

Beydoun, M. A., Beydoun, H. A., Noren Hooten, N., Maldonado, A. I., Weiss, J., Evans, M. K., & Zonderman, A. B. (2022). Epigenetic clocks and their association with trajectories in perceived discrimination and depressive symptoms among US middle-aged and older adults. *Aging (Albany NY), 14*(13), 5311-5344. doi:10.18632/aging.204150

Discacciati, A., Bellavia, A., Lee, J. J., Mazumdar, M., & Valeri, L. (2018). Med4way: a Stata command to investigate mediating and interactive mechanisms using the four-way effect decomposition. *Int J Epidemiol*. doi:10.1093/ije/dyy236

Kvamme, H., & Borgan, O. (2021). Continuous and discrete-time survival prediction with neural networks. *Lifetime Data Anal, 27*(4), 710-736. doi:10.1007/s10985-021-09532-6

Lewis, F. I., & Ward, M. P. (2013). Improving epidemiologic data analyses through multivariate regression modelling. *Emerg Themes Epidemiol, 10*(1), 4. doi:10.1186/1742-7622-10-4

Scutari, M., Denis, J.-B.,. (2022). *Bayesian Networks With Examples in R*. Boca Raton, FL: CRC Press.

VanderWeele, T. J. (2014). A unification of mediation and interaction: a 4-way decomposition. *Epidemiology, 25*(5), 749-761. doi:10.1097/EDE.0000000000000121
